# Improved cycle life and Li-ion transport parameters at low temperature in doped Ni-rich NMC cathodes

**DOI:** 10.1039/d6ta01388k

**Published:** 2026-04-23

**Authors:** Ethan Williams, David Burnett, Wilgner Lima da Silva, Jack E. N. Swallow, Ryan Parmenter, Robert S. Weatherup, Peter Slater, Emma Kendrick

**Affiliations:** a School of Metallurgy and Materials, University of Birmingham Edgbaston Birmingham B15 2TT UK e.kendrick@bham.ac.uk etw078@student.bham.ac.uk; b School of Chemistry, University of Birmingham Edgbaston Birmingham B15 2TT UK; c The Faraday Institution, Quad One, Harwell Campus Becquerel Avenue Didcot OX11 0RA UK; d Department of Chemistry, University of Manchester Oxford Road Manchester M13 9PL UK; e School of Physical Sciences, University of Kent Canterbury Kent CT2 7NH UK; f Department of Materials, University of Oxford Parks Road Oxford OX1 3PH UK

## Abstract

Ni-rich layered oxides such as (LiNi_*x*_Mn_*y*_Co_1−*x*−*y*_O_2_ (*x* ≥ 0.6)) exhibit structural degradation, surface instability, and poor lithium ion transport, particularly under extreme temperature conditions, limiting their viability for next generation high energy batteries. This work demonstrates that low-level boron (B25) and tin–boron codoping (SB25) enhance the structural resilience and electrochemical performance of LiNi_0.9_Mn_0.05_Co_0.05_O_2_ (NMC955) cathodes across a range of temperatures: −5 °C, 25 °C, and 45 °C. Both dopants integrate into the layered α-NaFeO_2_ structure, expanding lattice parameters and reducing cation mixing, while preserving particle morphology. At sub-ambient temperatures (−5 °C) where slow Li-ion transport is the primary limitation, Sn–B codoping delivers a 25% improvement in specific capacity at 500 mA g^−1^ relative to pristine NMC955, suppresses the emergence of a second high resistance charge transfer (*R*_CT_ reduces from 717 Ω to 71.4 Ω), and maintains the highest exchange current densities, 0.3 A m^−2^. At 25 °C *R*_CT_ is reduced, from 10.34 Ω in pristine NMC955 to 8.79 Ω, and the effective diffusion coefficient increases, from 1.5 to 1.6 × 10^−12^ cm^−2^ s^−1^, demonstrating enhanced low temperature transport kinetics. Long-term cycling at approximately 1C shows improved capacity retentions of 92.7% (B25) and 88.7% (SB25) after 100 cycles *versus* 78% for undoped NMC. Postmortem XPS/XAS confirm that codoping suppresses electrolyte-induced transition metal fluorination and CEI thickening, with Sn–B showing the smallest change in Ni oxidation state and local coordination after 200 cycles. Together, these results establish Sn–B co-doping as a scalable and effective strategy to simultaneously enhance the structural stability, interfacial chemistry, and low-temperature transport kinetics of Ni-rich NMC cathodes for demanding lithium-ion battery applications.

## Abstract 2

B and Sn–B co-doping of NMC955 enhances structural stability and Li-ion transport across −5 °C to 45 °C. Sn–B co-doping reduces charge-transfer resistance 8-fold at −5 °C and improves rate capacity by 25%, outperforming single-dopant strategies.

## Graphical abstract

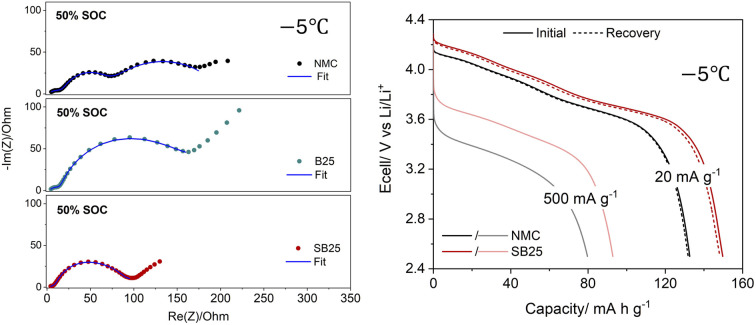

## 1 Introduction

One of the most significant challenges in adopting Ni-rich NMC cathode materials in next-generation electric vehicles (EVs) is their poor long–term cycle life.^1^ The origin of the declining capacity with increasing cycles has been attributed to structural degradation from cationic mixing disorder, irreversible phase transitions, microcrack evolution, surface reconstruction and oxygen release,^2–4^ as well as surface instabilities due to residual lithium compounds, electrolyte decomposition, and transition metal dissolution.^5–8^ To overcome these failure mechanisms, several strategies have been explored by researchers, such as bulk doping,^9–11^ surface coatings,^12–14^ electrolyte additives,^15–17^ and core–shell designed particles.^18,19^ Of these methods, bulk doping can be incorporated during the standard synthesis process and easily scaled to larger batch sizes. The role of the dopant when incorporated into the bulk structure has been previously attributed to providing strong M–O bonds, a pillaring effect to prevent detrimental phase transitions, and to expand the transition metal slab, thus improving lithium ion transport.^20,21^ Their inclusion has often contributed to extending the cycle life but at the expense of initial capacity due to their electrochemical/redox inactivity during the charge and discharge processes. When selecting a dopant, it is also important to consider elements of low cost, low criticality, and a robust supply chain so as not to impose additional economic and sustainability challenges when scaling-up the synthesis of the cathode active material.

The requirement for stable electrochemical performance over a wide temperature operation in lithium-ion batteries for EVs is another challenge for Ni-rich cathodes, which demonstrate poor thermal stability. The interfacial surface instability is exacerbated under elevated temperature testing, resulting in accelerated capacity degradation through reactions between the cathode and electrolyte. Studies on doped and surface-modified Ni-rich NMC cathodes have demonstrated improved high-temperature cycling stability by suppressing interfacial resistance growth, enhancing Li-ion diffusion, and stabilizing the crystal structure. Dopants such as Zr, Sn, Nb, and Ti, as well as protective coatings like Li_2_SnO_3_ and BaZrO_3_, have been shown to reduce cation mixing, limit side reactions, and strengthen particle integrity.^22–26^

The motivation for pursuing a Sn–B co doping strategy in Ni rich NMC stems from the complementary and mechanistically distinct roles played by the two dopants. Boron has emerged as a promising element for the doping in Ni-rich NMC, leading to enhanced electrochemical performance, either by promoting favourable radially aligned microstructures or forming a surface coating on primary crystallites, improving the cycle life.^27^ The B–O bond (806 kJ mol^−1^) is substantially stronger than any host TM–O bond (Ni–O 392, Mn–O 402, Co–O 368 kJ mol^−1^), providing a thermochemical driving force for framework stabilisation.^28^ Sn^4+^, has an ionic radius closely matching Ni^2+^, (ionic radius 0.69 Å in octahedral coordination, identical to Ni^2+^) and has also been identified as having a similarly stabilising effect on the structure due to the strong Sn–O bond (548 kJ mol^−1^).^28^ Sn^4^ (ref. 29 and 30) readily substitutes onto the transition metal 3a site and acts as a structural pillar, resisting displacement during delithiation and suppressing the destructive H2–H3 phase transition that drives microcracking in Ni-rich NMC at high state of charge.^29,30^ In addition, the low toxicity of Sn makes it a desirable choice for cation doping in lithium-ion cathode applications.^31^

Boron and tin dopants can also exert opposing but complementary influences on the lattice parameters. Sn^4+^ expands both the *a* and *c* axes due to its larger ionic radius relative to Ni^3+^/Ni^4+^, widening Li-ion diffusion channels. The incorporation of B, which is associated with lattice contraction or surface-layer formation depending on its site,^27^ provides a counterbalancing effect. Together, these effects yield a balanced and synergistic structural outcome not achieved by either dopant alone. This co-doping approach was therefore hypothesised to address the distinct failure modes of Ni-rich NMC under temperature extremes, enhancing structural stability at elevated temperatures while improving Li ion transport at sub-ambient conditions.

To this end, we have investigated how single and multi-element doping of boron, and boron and tin, can be employed in the synthesis of NMC 90-5-5 to improve the cycle life and understand their role in influencing the reaction kinetics of the cathode. Boron and tin–boron co-doping significantly improve the NMC structural stability, lithium-ion transport, and electrochemical performance across room, high (45 °C), and low (−5 °C) temperatures. Both doped materials retain the layered structure and show reduced cation mixing and improved lattice stability, while co-doped materials deliver the highest rate capability, highest Li-ion diffusivity, and lowest charge-transfer resistance, particularly under extreme temperatures.

## 2 Experimental

### 2.1 Synthesis of doped NMC 90-5-5

The [Ni_0.9_Mn_0.05_Co_0.05_](OH)_2_ precursor was initially synthesised by a hydroxide coprecipitation method, in which a 2 mol L^−1^ aqueous transition metal solution was first prepared from stoichiometric amounts of NiSO_4_·6H_2_O, MnSO_4_·H_2_O, and CoSO_4_·7H_2_O. The solution was then pumped into a 5 L CSTR reactor along with a pre-mixed solution of NH_4_OH (4 mol L^−1^) and NaOH (10 mol L^−1^) at a controlled rate to maintain a pH of 10.8. The reaction was maintained at 50 °C under a flowing N_2_ atmosphere for 8 hours, after which it was left to age for 2 days at temperature. The powder precursor material was, after vacuum filtration and washing, dried under vacuum at 120 °C overnight. The baseline NMC material was prepared by mixing 40 g of the precursor material with a 7.5% molar excess of LiOH on a roller mill for one day and subsequently fired in a tube furnace with a flowing oxygen feed at a rate of 0.5 L min^−1^, initially ramping to 450 °C at a rate of 1 °C min^−1^ for a 5 h hold and then to 750 °C to hold for 9 h. The doped materials in this study were prepared based on the target formula of Li([Ni_0.9_Mn_0.05_Co_0.05_]_1−*x*−*y*_B_*x*_Sn_*y*_)O_2_. In both cases, a target of doping 2.5% of the transition metal sites was used, where *x* = 0.025, *y* = 0 for B25 and *x* = *y* = 0.0125 for SB25. The required metal oxides (B_2_O_3_ and SnO_2_) were first ball milled for 3 h at 400 rpm before being mixed with the precursor powder and LiOH on the roller mill and fired at the same conditions as the baseline NMC.

### 2.2 Materials characterisation

Powder X-ray diffraction measurements were performed on a Proto AXRD Benchtop instrument with a Cu Kα source, in a 2*θ* range of 15–90° at a scan rate of 1° min^−1^. The particle morphology of the powdered cathode material was investigated using a Zeiss EVO10 scanning electron microscope using an accelerating voltage of 15 kV and a probing current of 50 pA. The elemental composition and valence of the particle surfaces were determined by X-ray photoelectron spectroscopy (XPS). The XPS measurements were performed using a PHI Versaprobe III XPS system with a monochromatic Al-Kα source (1486.6 eV). Samples were inertly transferred to the system from an Ar glovebox *via* a transfer vessel sealed with a rubber O-ring. All Core-level regions were measured at an analyser pass energy of 55 eV (providing a resolution of ∼0.55 eV, determined from fitting a Fermi function convoluted with a Gaussian to the Fermi edge of a sputtered Au foil) and energy step of 0.05 eV. Fitting of the XPS spectra was performed in the CasaXPS software, with the binding energies of each sample calibrated to the adventitious C 1s peak set to 284.8 eV, and a Shirley background was used for each spectra.

X-ray absorption spectroscopy (XAS) was collected at beamline B18 at the Diamond Light Source, UK, where measurements of the Ni, Co, and Mn K-edges were performed.^32^ The cathode powder materials were ground and diluted with PVP and pressed into 13 mm pellets that were then secured in Kapton tape and sealed in a vacuum pouch. Reference samples (>99%) were also purchased (Sigma Aldrich) and measured to aid with the calibration of the XANES oxidation state. X-ray absorption near-edge structure (XANES) spectra and Extended X-ray absorption fine structure (EXAFS) spectra were simultaneously collected in transmission and fluorescence modes. XANES and EXAFS data was extracted and analysed using the Athena software package, where the raw data was divided by the incident X-ray signal.^33^ The normalised EXAFS data were converted from energy (eV) to *k*-space with a *k*^3^ weighting, before a Fourier transformation into *R*-space and left uncorrected for photoelectron phase shifts.

Degradation studies were also performed and characterised by *ex situ* XPS and XAS experiments, in which spectra were collected on both the as-made electrodes and on electrodes after electrochemical cycling. Half-cells were constructed in two electrode Swagelok cells and cycled 200 times between 4.3–2.5 V with a charge current density of 100 mA g^−1^ and discharge current density of 200 mA g^−1^. The cells were disassembled in a glovebox, and the electrode disc was washed in dimethyl carbonate to remove the residual electrolyte before sealing under the glovebox atmosphere. *Ex situ* XRD was collected on a Bruker D8 Advance diffractometer (Cu Kα1,2) between 15–90° 2*θ* (step size 0.0189 and 253.3 s per step). Electrodes were loaded onto a domed sample holder (Anton Paar) inside an argon filled glovebox. For investigation of the 1st cycle, cells were disassembled and cleaned as stated above following an initial rest at OCV for 6 h and subsequent constant current–constant voltage (CC–CV) charging to 4.3 V at 10 mA g^−1^ or after the following cc discharge to 2.5 V at the same current density. *Ex situ* XRD was also performed on electrodes extracted from cells at the same voltage cut-offs following asymmetric cycling with cells charged/discharged at 200/500 mA g^−1^.

### 2.3 Electrochemical testing

Electrode coatings were prepared by mixing the cathode active material, PVDF binder solution, carbon black, and synthetic graphite in a 90 : 5 : 3 : 2 weight ratio into a homogeneous slurry which was then coated onto an aluminium current collector. Electrodes were dried overnight at 120 °C and assembled into CR2032-type coin cells using 1 M LiPF_6_ in EC/EMC (3 : 7 by volume) + 2 wt% VC for the electrolyte solution with a Celgard separator and a lithium metal counter electrode. All tested half-cells underwent the same formation process after an 8 h rest to allow for complete electrolyte wetting, using two galvanostatic charge/discharge cycles at an applied current density of 10 mA g^−1^ at a voltage range of 4.3–2.5 V.

Rate capability was tested at a constant charging current density of 20 mA g^−1^ and a variable discharge current density of either 20, 50, 100, 200, 500, 1000, and 2000 mA g^−1^ for 5 cycles at each rate. Electrochemical impedance spectroscopy (EIS) was performed at various voltages after a 30 min rest over a frequency range from 100 kHz to 10 mHz at an amplitude of 10 mV. The evaluation of the exchange current density (*J*_0_) requires an estimation of the particles' electrochemically active surface area (S) using eqn (1), for which a spherical geometry is assumed.1
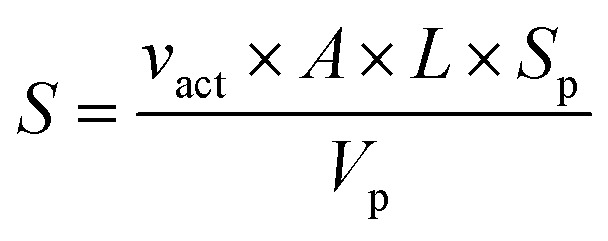
where *v*_act_ is the active material volume fraction in the electrode, *A* is the geometric area of the electrode disc, *L* is the electrode thickness, *S*_p_ is the surface area of a sphere using the measured mean particle diameter from the particle size analysis, and *V*_p_ is the volume of the same sphere. The exchange current density (*J*_0_) can then be calculated from this value of *S* by a linearized form of the Butler–Volmer equation as shown in eqn (2).2
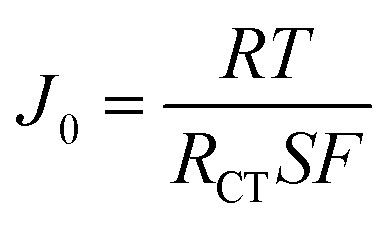
with *R* as the ideal gas constant, *T* as the testing temperature (in K), and *F* as the Faraday constant.^34^ The exchange current densities are also presented at varying lithium stoichiometries (*x*_Li_) which can be calculated at different SOC by the relationships in eqn (3) and (4)3
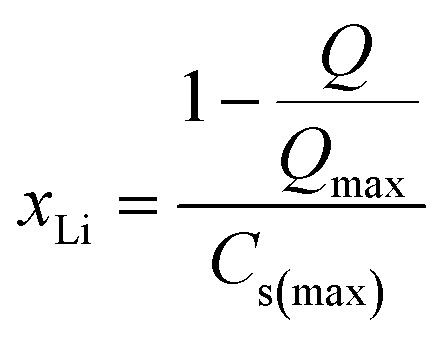
4
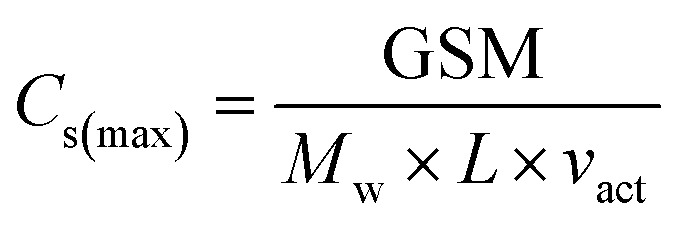
where *Q* is the capacity at a particular SOC, *Q*_max_ is the discharge capacity at 100% SOC, and *C*_s(max)_ is the maximum lithium concentration in an electrode, calculated from its areal loading, GSM (in g m^−2^), molecular weight of the active material, *M*_w_, the electrode thickness, *L*, and the active material volume fraction, *v*_act_. Note a stoichiometry of *x*_Li_= 0 is never achieved in these cathode materials due to the inability to remove all the lithium from the layered structure as this is prevented by thermodynamics and structure collapse.

Galvanostatic intermittent titration technique (GITT) tests were performed by applying transient current pulses at a rate of C/15 for 15 min followed by a 2 h relaxation period. Diffusion coefficients were calculated based on the Weppner and Huggins method using eqn (5), in which the SAND equation is applied to fit the individual current pulses, as described in previous work.^35^5
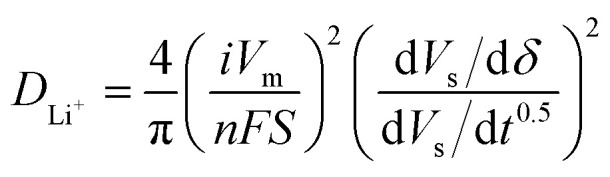
*i* is the current, *V*_m_ is the molar volume, *n* no. of electrons per formula unit, *F* is the Faraday constant, d*V*_s_/d*δ* is the change in the electrode potential with respect to lithium stoichiometry, and d*V*_*I*_*/*d*t*^0.5^ is the gradient of the voltage response during the current pulse with respect to *t*^0.5^. To investigate the effect of temperature extremes, the electrochemical testing described above was also performed at temperatures of 45 °C and −5 °C, in which cells were placed inside an environmental test chamber (ECO HTLC135, Temperature Applied Sciences Ltd) after undergoing formation at room temperature. Before the start of each test at a new temperature, a 3 h rest step was instigated to allow for thermal acclimatisation.

## 3 Results and discussion

### 3.1 Optimised doping

**Fig. 1:**
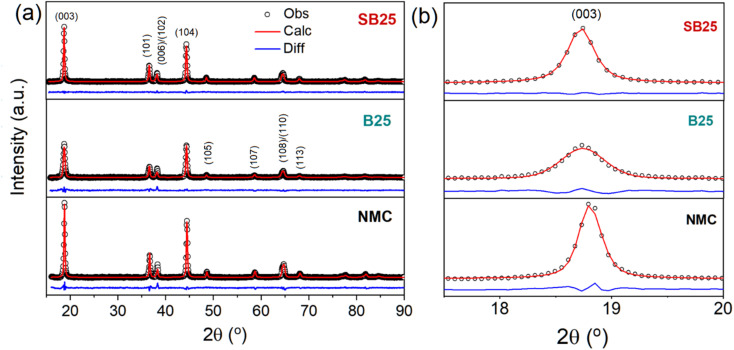
Observed, calculated, and difference profiles from Rietveld refinements using powder XRD data for the baseline and doped NMC 90-5-5 materials (a), and the inset of the (003) peak (b).

The XRD patterns in Fig. 1 show retention of the R-3m layered α-NaFeO_2_-type rock-salt structure is observed for the synthesis of the boron doped (B25) and tin–boron doped (SB25) NMC-90-5-5 materials. No additional peaks are present for the doped material, indicating the phase purity and supporting the incorporation of the doped cations into the bulk material. The doping is also evident from the shift in the (003) diffraction peak to lower angles compared to the pristine NMC, often attributed to an expansion in the *c* lattice parameter.^36,37^ Furthermore, the peak broadening is increased in the doped materials which is usually indicative of a smaller primary particle size and an increased number of grain boundaries within the secondary particles.^38^

Rietveld refinements were also performed using the XRD data to elucidate further information about the influence of doping on the NMC lattice parameters, which are presented in Table 1. The lattice parameters *a* and *c*, and the lattice volume all increase for the doped materials, further supporting the successful incorporation of the cations into the crystal lattice. The calculated *c* parameter is greater in SB25 which is consistent with the presence of the larger Sn^4+^ cation in the layered structure. The cation mixing between Ni^2+^ and Li^+^ in NMC is typically assessed by the intensity ratio of the (003) to (104) peaks, where a value lower than 1.2 is usually associated with a considerable extent of mixing. The values of the peak intensity ratios were calculated as 1.32, 1.05, and 1.47 for NMC, B25, and SB25 respectively, which would indicate that SB25 demonstrates the lowest proportion of cation mixing. However, the calculated Ni on the Li site from the Rietveld refinement is slightly larger in SB25 than for the pristine NMC. This could suggest the presence of some Sn in the Li layer, which has previously been observed in Li-rich NMC due to the similar ionic radius of Sn^4+^ (0.71 Å) and Li^+^ (0.76 Å).^39^ The larger value of *I*(003)/(104) in SB25 could therefore be due to the higher scattering factor of Sn compared to Ni and thus a larger contribution to the peak intensity at this site.

**Table 1:** The lattice parameters of the pristine and doped NMC 90-5-5 materials are calculated from Rietveld refinement

Sample	*a*/Å	*c*/Å	Volume/Å^3^	Density/g cm^−3^	Ni on Li	Rw_p_%^a^
NMC	2.87556(9)	14.1860(6)	101.586(6)	4.709	0.028(2)	5.29
B25	2.8811(1)	14.205(1)	102.11(1)	4.874	0.082(1)	5.02
SB25	2.88103(7)	14.2140(4)	102.174(4)	4.883	0.067(1)	2.01

a NB. The weighted profile *R*-factor (Rw_p_, %) is a standard goodness-of-fit metric for Rietveld refinements.

**Fig. 2:**
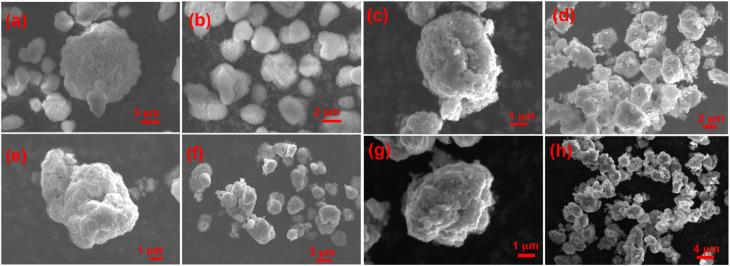
SEM images of the Ni_0.09_Mn_0.05_Co_0.05_(OH)_2_ precursor material (a) and (b), and the cathode active materials of the undoped NMC (c) and (d), and doped B25 (e) and (f), and SB25 (g) and (h).

Scanning electron microscopy (SEM) was used to assess the morphology of the precursor material before firing and the subsequent active materials of the pristine and doped NMC 90-5-5 materials, which are shown in Fig. 2. The precursor morphology consists of sharp platelets that have agglomerated into quasi-spherical secondary particles. The SEM images of the cathode active materials reveal that these platelets are fused into denser cuboidal primary particles that are packed into denser secondary particles. Bulk doping with either boron or tin and boron shows no significant observable change in the particle morphology of the NMC material. Particle size analysis of the cathode materials revealed a smaller median (D50) particle size in the doped materials of 4.19 µm and 5.48 µm for SB25 and B25, compared with 5.96 µm in NMC.

XPS was conducted on the active powder materials to investigate the effect on the chemical state and surface composition in the pristine and doped NMC cathodes. The B 1s, Sn 3d, and Ni 2p spectra are displayed in Fig. S1. The main Ni 2p_3/2_ and 2p_1/2_ peaks in SB25 are positioned at 854.9 eV and 872.4 eV, respectively, consistent with the undoped NMC material, with a small shift to 855.5 eV and 872.7 eV observed for B25. In all cases, the Ni 2p_3/2_ peak positions are at slightly higher binding energies than those typically assigned to Ni^2+^ species. Whilst the Ni 2p has a complex lineshape related to multiplets and satellites^40^ making charge determination difficult with XPS, we tentatively suggest that the oxidised nickel is likely present in a mixed Ni^2+^/Ni^3+^/Ni^4+^ oxidation state in each of the cathode materials, consistent with that seen for other pristine Ni-rich NMC cathodes *via* Ni L-edge XANES previously.

The presence of Sn at the surface of SB25 is confirmed by the characteristic Sn 3d_5/2_ and 3d_3/2_ peaks at 485.3 eV and 493.7 eV, displaying the expected spin–orbit splitting for Sn 3d, and peak positions generally consistent with Sn^4+^ doping as previously reported in adjacent NMC materials.^39,41,42^ The B25 and SB25 materials also both show a single peak at 191.5 eV and 190.8 eV in their respective B 1s spectra. The peak is positioned at lower binding energy than that characteristic of B_2_O_3_ (193–194 eV) and instead is more aligned with the presence of lithium borate compounds.^43,44^ This could suggest some reaction between the B_2_O_3_ and the LiOH during the calcination step and subsequent formation of a Li_3_BO_3_ or LiBO_2_ layer at the surface. Together, the B 1s and Sn 3d spectra indicate the successful incorporation of the dopants into the NMC structure, with no residual unreacted phases present in B25 or SB25.

**Fig. 3:**
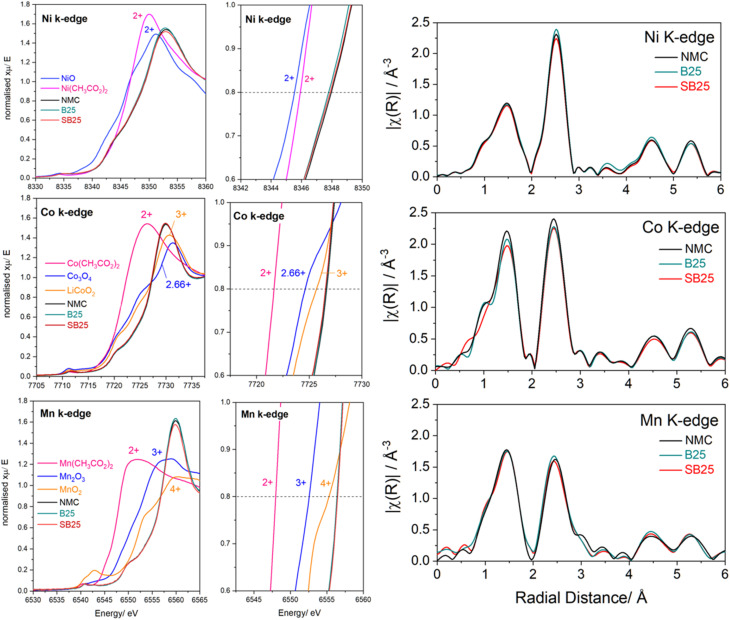
Normalised XANES spectra of the baseline and doped NMC cathode materials as well as the reference compounds at the Ni, Co, and Mn K-edges. EXAFS spectra of the cathode materials at the same metal edges.

To further probe the structure and oxidation states of the NMC transition metals in the bulk materials, XANES measurements were carried out on the cathode powders at the Ni, Co, and Mn K-edges. The resulting XAS spectra are displayed in Fig. 3, along with the measured standard Ni, Mn, and Co reference compounds of known oxidation states. The values of the threshold energy (*E*_0_) were measured following normalisation of the pre-edge and post-edge regions and are compared for the standards and samples at the different metal K-edges in Table S1.

The *E*_0_ values determined from the position of the peak maximum (white line energy) at the Ni K-edge for the NMC and doped samples are all located at ∼8352 eV, displaying a shift to higher energy compared to the nickel(ii) acetate (8349.1 eV) and nickel(ii) oxide (8350.4 eV) standards. This suggests the presence of Ni in a higher oxidation state than Ni^2+^ in each of the cathodes. The difficulty in finding a reliable standard consisting of purely Ni^3+^ or Ni^4+^ due to their instability towards reduction or disproportionation, and so a complete assessment of the Ni oxidation state could not be acquired.^45,46^ Overall, the similar peak position and shape of the XANES spectra of the doped materials at the Ni edge closely align with the pristine NMC, suggesting the bulk structure is preserved upon the addition of boron and tin.

Inspection of the Mn K-edge *E*_0_ values reveals that all the cathode materials are at higher energies than the manganese(iv) oxide standard. The absorption peaks of the cathode materials in the Co K-edge were within <1 eV of the peak from the Co^3+^, indicating Co is present in the trivalent state for each of the materials. These results agree with the theoretical oxidation states of Mn^4+^ and Co^3+^ in NMC, as well as the valence states and XANES curves seen in the literature for Ni-rich NMC cathodes.^47,48^ The difference in edge energy determined from the white line positions in each of the metal K-edges are all less than 0.5 eV for NMC, B25, and SB25, which suggests doping does not result in a significant level of charge compensation.

The pre-edge region of the XANES can give information regarding the metal's coordination and environment. The presence of a small pre-edge peak in the Ni K-edge centred just below 8335 eV can be observed in all of the cathode samples. This pre-edge feature is usually attributed to the hybridisation between dipole-forbidden Ni d orbitals with O p and Ni p orbitals.^49^ The pre-edge peak is also observed in the Ni^2+^ standards at a similar energy of 8334.3 eV. Close inspection of the Mn pre-edge region revealed the presence of two peaks at 6541 eV and 6543 eV, which is consistent with the 1s to 3d electronic transitions associated with octahedral Mn^4+^ that has previously been reported in different NMC compositions.^48^

Data were also collected in the full EXAFS region for each of the materials to elucidate whether the introduction of the dopants resulted in changes in the local coordination environment of the NMC transition metal elements (Fig. 3). The Ni EXAFS data show two main peaks for the cathode materials at distances of 1.47 Å and 2.52 Å relating to the scattering between Ni–O and Ni–TM nearest neighbour interactions. The position of these peaks in the pristine and doped NMC show close overlap, indicating no distinct change in the bond lengths are observed upon B or Sn modification. However, the peak intensity of the Ni–TM environment slightly increases in B25 but decreases in SB25 relative to the baseline NMC, which can be correlated positively with changes in coordination number.^50^ A similar trend is seen in the Mn spectra, which overall shows little variation between the baseline and doped NMC cathodes, suggesting the Mn environment is mainly retained. For Co, the doped materials show slightly lower populations in both the Co–O and Co–TM coordination, however the change is relatively small and not observed in the other transition metal EXAFS, so it is unlikely that oxygen vacancies are present.

A combined analysis of the XRD, XPS, and XAS/EXAFS data provides clear evidence for the successful incorporation of boron and tin into the NMC955 structure and elucidates the mechanisms through which these dopants enhance bulk and surface stability. Sn^4+^ substitution expands and reinforces the transition metal layers, reducing cation mixing and suppressing the lattice collapse associated with the H2–H3 phase transition, while boron contributes both to bulk lattice stiffening and to the likely formation of a stabilising lithium borate surface layer that mitigates electrolyte-induced degradation. The precise location of B in the NMC lattice is genuinely contested in the literature, there are three main hypotheses: (i) substitution onto the octahedral transition-metal site (3a Wyckoff position). (ii) Occupation of tetrahedral interstitial sites in the Li layer, as supported by DFT calculations^51^ and confirmed by neutron diffraction in NCM811 by Ying *et al.*,^52^ who showed that B preferentially incorporates into tetrahedral interstices in the Li layers rather than substituting any specific atom. (iii) Segregation to the particle surface as a thin Li–B–O layer (Li_2_BO_3_ or LiBO_2_), as argued by Skvortsova *et al.*^53^ on the basis of DFT and EELS evidence showing complete insolubility of B in the LiNiO_2_ structure. A further view, from Roitzheim *et al.*^54^ and supported by ion beam analysis and TOF-SIMS, is that boron shows trigonal planar or tetrahedral coordination in the Ni layers with an inductive TM–O–B effect that delays structural collapse. In this work, the XPS data indicates a likely lithium borate surface layer, with additional doping into the bulk. Due to the low levels of dopants, a precise crystallographic position is difficult to elucidate. HRTEM, neutron diffraction or EELS would provide a complementary atomic-resolution view and is a valuable direction for future work.

### 3.2 Electrochemical performance

The galvanostatic capacity profiles of the 1st and 2nd cycles recorded at a 10 mA g^−1^ rate within a voltage range of 4.3–2.5 V are shown in Fig. S2 from triplicate coin cell data for the pristine and doped materials. The recorded first cycle loss (FCL) was below 10 mAh g^−1^ for each material, whilst the coulombic efficiencies (CE) were >98%, reflecting excellent structural stability during the creation of the CEI and decomposition of the electrolyte.^55^ The SB25 material demonstrates the highest electrochemical reversibility in the half cells, with a FCL of 5.1 mAh g^−1^ and a FCE of 97.6%. The shape of the capacity profile is largely unaffected by the bulk doping, however a slight increase in the polarization below 3.4 V is observed in B25, whilst the upper voltage sloping profile usually present in Ni-rich NMC materials above 4.2 V has been supressed. This is reflected in the lower discharge capacity of the doped B25 in the first cycle of 190.8 mAh g^−1^ compared to those of NMC (208.0 mAh g^−1^) and the co-doped SB25 (210.2 mAh g^−1^). The lower initial specific capacity of B25 is also consistent with the earlier diffraction results which suggests B25 demonstrates the largest proportion of cationic mixing, which can limit the amount of available Li^+^ during charge and discharge.

**Fig. 4:**
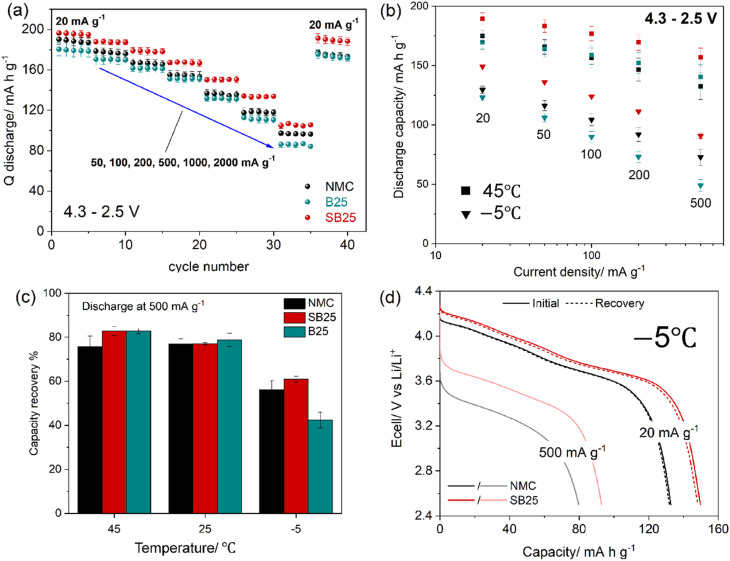
Rate capability of pristine and doped NMC materials at room temperature (a) and at 45 °C and −5 °C (b). Rate capacity recovery at 500 mA g^−1^ at different temperatures (c) and selected discharge profiles of the pristine and SB25 doped NMC at −5 °C (d).

The rate performance of the pristine and doped materials was assessed by asymmetric cycling with increasing discharge rates, using current densities between 20–2000 mA g^−1^ in a voltage range of 4.3–2.5 V. As displayed in Fig. 4, SB25 delivers the highest specific discharge capacities over the five cycles recorded at each of the tested rates, demonstrating the largest increase at 500 and 1000 mA g^−1^ where larger initial capacities of 150 mAh g^−1^ and 134 mAh g^−1^ were obtained, compared with 137 mAh g^−1^and 118 mAh g^−1^ in the pristine NMC. However, when the applied current density was increased to 2000 mA g^−1^, the capacity gain is less significant, with SB25 and pristine NMC delivering 105 mA h g^−1^ and 97 mA h g^−1^ respectively. The specific capacities of B25 were instead slightly lower than the pristine NMC at each of the discharge rates, delivering initial capacities of 131 mA h g^−1^ and 113 mA h g^−1^ at applied current densities of 500 and 1000 mA g^−1^. However, B25 demonstrated a higher retention of its initial capacity at 20 mA g^−1^ than NMC at current densities up to 1000 mA g^−1^, whilst SB25 delivered the highest capacity retention at all rates, demonstrating its superior rate performance. Following the fast discharge rates, recovery cycles were performed at 20 mA g^−1^, yielding final capacity retentions of 91%, 95%, and 96% for NMC, B25, and SB25 respectively, illustrating the benefits of the doping.

The rate capabilities of the three cathode materials were also investigated at temperatures of 45 °C and −5 °C at current densities between 20 and 500 mA g^−1^ to probe the effect of doping on the Li-ion kinetics at non-ideal extreme conditions. The temperature varied rate performance of the different cathodes are shown in Fig. 4, and largely reflect the trends observed at room temperature testing, with SB25 demonstrating higher discharge capacities for each rate when cycled at 45 °C or −5 °C. However, the rate capability of B25 relative to the pristine NMC is considerably better at 45 °C than at −5 °C, delivering higher specific capacities than NMC at current densities of 100 mA g^−1^ and above. The rate capacities obtained at 45 °C for each cathode are similar in magnitude to those recorded at room temperature, but in the case of the doped materials, higher values were observed at the elevated temperature when cycled at the faster rates. The discharge capacities obtained at 500 mA g^−1^ were 133, 140, and 157 mAh g^−1^ for NMC, B25, and SB25, corresponding to 76%, 83%, and 83% of their initial 45 °C rate capacity (at 20 mA g^−1^), compared to 71%, 73%, and 77% at room temperature. These results indicate that the doped materials are effective in enhancing the rate performance at room temperature and, more significantly, at elevated temperatures.

The specific capacities obtained from the rate capability tests performed at −5 °C were noticeably lower than those recorded at room temperature for both pristine and doped NMC. As seen at higher temperatures, SB25 shows the highest specific discharge capacities, delivering 149, 136, 124, 111, and 91 mAh g^−1^ at current densities of 20, 50, 100, 200, and 500 mA g^−1^ compared to 130, 117, 104, 92, and 73 mAh g^−1^ in the pristine NMC tested at the same rates. Contrasting to its rate performance at higher temperatures, the capacity delivered by B25 at −5 °C remained lower than that of the pristine material at faster rates. At 500 mA g^−1^, B25 delivered only 42.4% of its initial rate capacity, compared to 56.2% for the pristine NMC. However, in the following recovery cycles at 20 mA g^−1^, B25 and pristine NMC both recovered 98% of their initial rate capacity, while the SB25 showed an even higher 99% capacity return.

**Fig. 5:**
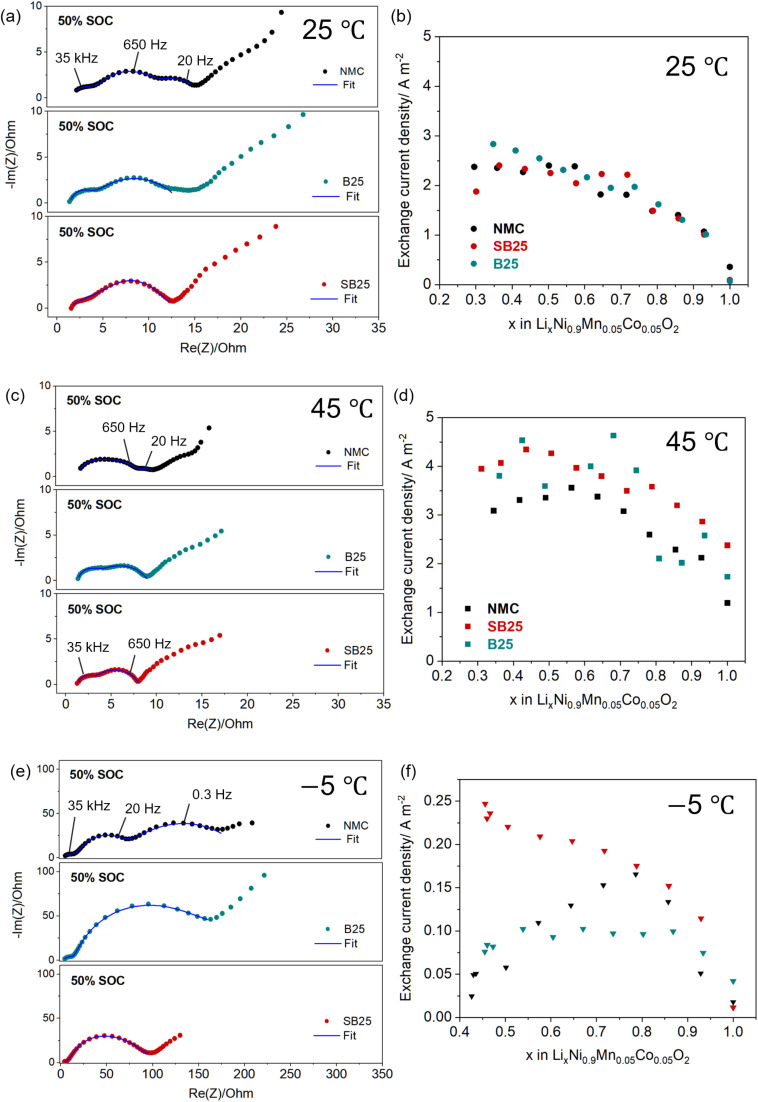
Nyquist plots from the EIS measurements at 50% SOC at various temperatures (a, c, e) and the calculated exchange current densities as a function of Li stoichiometry during charge (b, d, f).

EIS measurements were performed on the pristine and doped materials to evaluate the effect of doping on the electrode–electrolyte interfacial resistances. The impedance spectra were collected on fresh cells after formation at room temperature at different states of charge (SOC) between 2.5 and 4.3 V. Fig. 5 shows the fitted Nyquist plots of the three cathodes at 50% SOC which all share a small semi-circle at around 100 kHz, assigned to the cathode electrolyte interface (*R*_SEI_), followed by a larger semi-circle centred at 0.5 kHz which is attributed to the charge transfer resistance (*R*_CT_). In the pristine NMC material, the charge transfer semi-circle in the medium to low-frequency region is split into two, with a further semi-circle appearing at 0.02 kHz. This additional semi-circle is subdued for B25 and disappears completely for SB25. The depression of this additional resistive process in the doped cathodes and their improved rate capability suggest the dopants act to provide further surface stabilisation by forming a conductive layer. The *R*_CT_ values at 50% SOC for NMC, B25, and SB25 are 10.34 Ω, 10.04 Ω, and 8.79 Ω, revealing the combined boron and tin doping is more effective in reducing the charge transfer resistance at the mid charge point. The ohmic resistance (*R*_S_) values in the B25 and SB25 at 50% SOC are 1.13 Ω and 1.42 Ω, which are lower than the 4.08 Ω calculated for the pristine NMC. The doped materials also showed smaller *R*_S_ values during the entire charge cycle, as listed in Table S2, suggesting that doping B^3+^ and Sn^4+^ into the NMC structure reduces the internal resistance of the electrolyte.

The temperature dependence of the impedance in the pristine and doped NMC cathodes was also studied by EIS measurements at 45 °C and −5 °C, for which the Nyquist plots at 50% SOC are shown in Fig. 5. At 45 °C, the additional lower frequency semi-circle is no longer present in the impedance spectra of the pristine NMC. Instead, it displays an elongated semi-circle in the high frequency region followed by a second smaller semi-circle at 0.2–0.02 kHz. The shape of the Nyquist plots of B25 and SB25 are more consistent with those at room temperature, except for the smaller diameter of the semi-circles. The *R*_CT_ values of at 50% SOC are quite similar between the pristine NMC and the doped materials, being 5.9 Ω compared to 4.5 Ω and 5.5 Ω for B25 and SB25. However, at 100% SOC, the *R*_CT_ value for the pristine NMC increases considerably to 20.2 Ω, whereas no increase is observed in the doped cathodes, further highlighting the improvements on doping. Additionally, the SEI resistance measured in the cells at 45 °C is lower for B25 and SB25 over the complete SOC range.

The EIS responses of the cathodes recorded at −5 °C display much larger semi-circles in the Nyquist plots corresponding to greater impedance across the cell. The increased resistance at low temperatures is thought to be associated with slower ionic and electronic transport within the solid electrode, the electrolyte, and at the CEI, resulting in large overpotentials.^56,57^ The Nyquist impedance of the pristine NMC at −5 Ω recorded at 50% SOC again shows the distinct appearance of an additional semi-circle in the low frequency region, which is still absent in the spectra of the doped cathodes. This is further demonstrated in the Bode plots displayed in Fig. S3, which shows the two low frequency signals for NMC at ∼10^−1^ Hz and ∼10^1^ Hz are replaced by a single signal in this region in the doped samples. However, compared to the appearance at room temperature, the third semi-circle appears to be the dominant impedance process in the pristine NMC, which is supported by the fitting results, which returned *R*_CT_ values of 50.7 Ω and 136 Ω for the two larger semi-circles. The impedance profile of the single and multi-cation doped NMC cathodes at low temperatures are similar in shape, displaying a single charge transfer. However, the SB25 material demonstrates a considerable reduction in the charge transfer impedance, with an extracted *R*_CT_ value at 50% SOC of 86.5 Ω, compared to 172 Ω for B25 which is similar to the combined *R*_CT_ values in the pristine NMC. The stabilisation of the charge transfer resistance in SB25 is further demonstrated at higher SOC, in which it decreases slightly to 71.4 Ω when charged to the upper voltage limit. In the pristine NMC, the first *R*_CT_ process remains constant with increasing SOC at around 50 Ω, however the second *R*_CT_ process grows considerably to 356 Ω at 4.2 V and finally to 717 Ω when charged up to 4.3 V.

To further understand the reaction kinetics, the exchange current densities were calculated at each SOC according to the Butler–Volmer relationship from the *R*_CT_ values obtained from the EIS fitting and are then normalised by the active electrochemical surface area. Fig. 5 displays the evolution of *J*_0_ as a function of lithium stoichiometry in the pristine and doped NMC materials at the various temperatures. At room temperature, the exchange current densities of the baseline and doped materials all follow a parabolic-like trend as a function of delithiation, with values ranging from 1.0 A m^−2^ and 2.7 A m^−2^ which is consistent with values previously reported for Ni-rich NMC811 materials.^58,59^

At the elevated temperature of 45 °C, the calculated values of *J*_0_ in all the cathodes increase, with these values now in the range of 2.0 A m^−2^ and 5.6 A m^−2^. The evolution of *J*_0_ with decreasing lithium stoichiometry follows a similar curve as that observed at room temperature, however the exchange current densities are generally higher in the doped cathodes over the majority of the stoichiometry range. The average values of *J*_0_ at 45 °C are reported as 2.6, 3.6, and 3.5 A m^−2^ for NMC, SB25, and B25 respectively, suggesting improved kinetics in the doped materials at higher temperature. The exchange current densities drop significantly at −5 °C in all cathodes to values below 0.3 A m^−2^, whilst the smaller stoichiometry range reflects the lower amount of available lithium that can be extracted at the sub-zero temperature. The inset in Fig. 5 reveals a larger variation in the behaviour of *J*_0_ between the different cathode materials at −5 °C, with the exchange current densities in the baseline NMC following an inverted V-shape, reaching a maximum of 0.166 A m^−2^ at 30% SOC. Conversely, the exchange current density in B25 displays a plateau above 20% SOC at a value close to 0.1 A m^−2^ for the rest of charge window, whilst the evolution of *J*_0_ at increasing SOC in SB25 is more consistent with the behaviour seen at higher temperatures. Notably, SB25 exhibits the highest values of *J*_0_ across the full delithiation process at the low temperature conditions, displaying considerable improvement compared to the baseline NMC at higher SOC.

GITT measurements were performed in coin cells after the formation process to extract information on the diffusivity of lithium ions across the different NMC materials. Fig. S4 shows the effective diffusion coefficients for the pristine and doped NMC materials measured at different temperatures. At room temperature, the introduction of the boron and tin dopants does not have a significant effect on the shapes of the diffusion curves, whilst the diffusion profiles of each material measured at 45 °C are largely consistent with those at room temperature. However, at −5 °C a larger variation in the effective diffusion coefficients is revealed across the complete charge and discharge cycle, displaying a considerable drop off towards the fully de-lithiated and lithiated states. The variation in the effective diffusion coefficients between the different cathode materials is also more obvious at this temperature, with SB25 demonstrating a larger *D*_Li^+^_ over the full voltage window, most notably when the cathode is charged between 3.8 V and 4.3 V. This is also demonstrated in Fig. S4 which displays the average charge and discharge *D*_Li^+^_ values for each cathode at the different temperatures, which generally follow the trend of SB25 > B25 > NMC. Additionally, it was found that the cells tested at room temperature displayed the highest average charge/discharge *D*_Li^+^_ values of 1.50/1.57, 1.51/1.44, and 1.62/1.66 × 10^−12^ cm^−2^ s^−1^ for NMC, B25, and SB25 respectively. This result is counterintuitive as higher temperatures typically enhance the solid-state diffusion of Li^+^ in the active material. Despite leading to faster kinetics, as seen in the EIS results, lithium-ion transport within the solid cathode material may be hindered due to structural changes arising at 45 °C, such as phase changes or cation mixing which reduce Li^+^ mobility within the cathode structure.

### 3.3 Dopant stabilisation

**Fig. 6:**
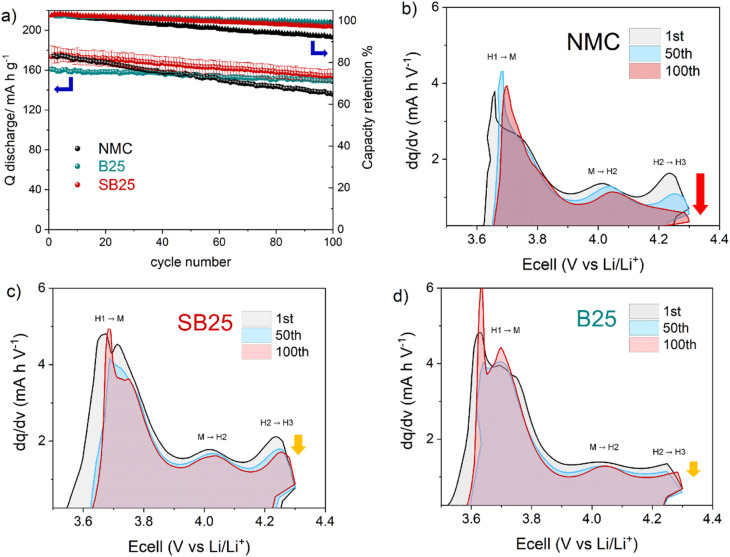
Cycle performance of NMC, B25, and SB25 measured between 4.3–2.5 V at 200 mA g^−1^ (a) and the corresponding d*Q*/d*V* curves of the 1st, 50th, and 100th charge cycle for pristine NMC (b), SB25 (c), B25 (d).

To assess the structural stability of the materials under battery operation, cycling tests were performed with a discharge rate of 200 mA g^−1^ (≈1C). As seen at the slower rate, the initial capacity of the NMC and SB25 are higher than those delivered by B25, however the capacity fade is much slower in the boron doped material over the 100 cycles. After 50 cycles the discharge capacity of NMC falls below B25, whilst that delivered by SB25 still remains the highest for the duration of the cycle test. The capacity retentions of the materials are tracked along with the discharge capacity in Fig. 6, and are higher for both the doped materials with final values of 92.7% (B25) and 88.7% (SB25) compared to 78% in the pristine NMC.

The effect of bulk cation doping on extending the cycle life can be further understood by assessment of the sequential d*Q*/d*V* plots, which track the evolution in phase transitions for the 1st, 50th, and 100th cycle in Fig. 6. The NMC materials all show three peaks relating to the main phase transitions associated with the layered oxide materials during lithiation/de-lithiation. Inspection of the H2–H3 peak in the pristine NMC reveals a large decline in intensity between the 1st and 100th cycle, reflecting the poor reversibility in continuously forming the H3 phase in the highly de-lithiated state. In contrast, the loss of the H3 phase is significantly negated in the SB25 sample, showing a higher retention of the high voltage peak over the cycle range, which reflects the higher capacity retention. This is also the case for B25, however the initial peak intensity is much smaller for this material, suggesting a smaller proportion of the H3 phase is formed originally which is reflected in the greater degree of Li/Ni mixing and lower capacity of this material, as these phase transitions are strongly related to the lithium stoichiometry.^3^ The suppression of the H2–H3 phase transition brought about by the singular boron doping will also reduce the large contraction of the crystallographic *c*-axis, which is associated with this transition and contributes heavily to the formation of microcracks.^60^ Postmortem SEM images after 200 cycles are shown in Fig. S8 and S9.

### 3.4 *Ex situ* XRD

The evolution of the crystal structure upon electrochemical cycling was investigated by measuring the *ex situ* XRD patterns of the pristine and doped NMC cathodes after being charged to 4.3 V or following the subsequent discharge to 2.5 V. Fig. S5 compares the diffraction patterns of the as made electrodes with those at the end of charge and discharge of the first cycle and at the same voltage points in cells after formation and an additional 20 cycles at 200/500 mA g^−1^. Compared to the XRD of the active material powders, the diffraction patterns of each of the as made electrodes show additional peaks at 26.6° and 65.1°, which are attributed to the presence of graphite added in the electrode formulation and the aluminium foil respectively. During the first electrochemical cycle, additional low intensity peaks appear in the 2*θ* range between 20–25° which are consistent with previous *ex situ* XRD studies of NMC materials and are related to superlattice peaks arising from cation ordering between Li and TM ions as well as the presence of an active monoclinic (*C*2/*m*) phase.^61–63^ The splitting of the (108)/(110), which is indicative of a well layered structure, also remains consistent between the first cycle and in the cycled cells, suggesting the layered structure is largely unaffected by the fast rate cycling.

**Fig. 7:**
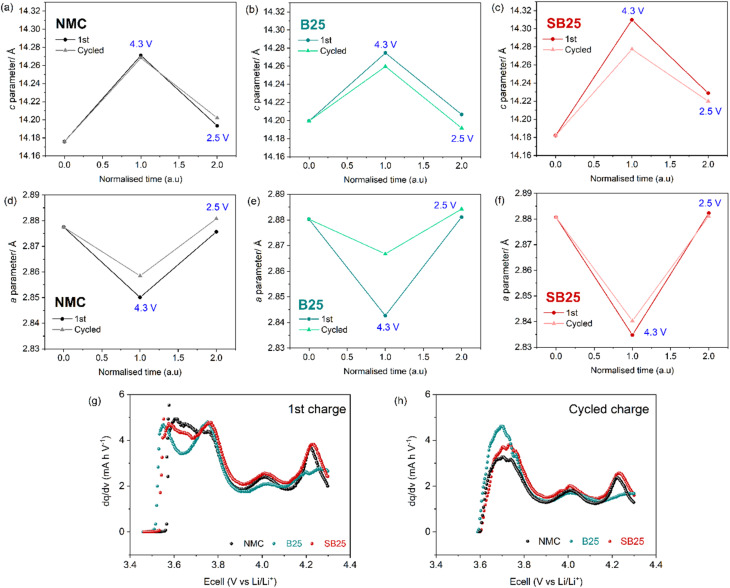
Changes of the unit cell parameters calculated from refinements of the *ex situ* XRD patterns (a–f) collected on electrodes from cells cycled between 4.3–2.5 V at 10 mA g^−1^ during the first formation cycle and after 20 cycles at a discharge rate of 500 mA g^−1^, and the corresponding d*Q*/d*V* plots of the charge cycle in each cell (g and h).

Rietveld refinements were also performed on the diffraction patterns from the *ex situ* XRD studies to quantify the changes in structure following (de-)lithiation. The resulting variations in unit cell parameters upon each charge or discharge cycle are presented in Table S6 and Fig. 7. In all the cathodes, an increase in the *c*-parameter and decrease in the *a*-parameter is observed at the end of charge relative to its length in the pristine electrode. The change in the *c*-axis is also reflected in the shift in the 2*θ* position of the (003) peak to lower angles and is indicative of a contraction in the interlayer spacing due to the removal of lithium from the Li layer, enabling an increase in electrostatic repulsions between oxygen in adjacent layers.^64^ Conversely, the shrinking of the *a*-axis is a result of the increase in Ni oxidation state from the de-lithiation of the cathode, leading to stronger and shorter Ni–O bonds which reduces the average TM–TM interatomic distance. As the cathode materials undergo lithiation in the discharge, the *a* and *c* parameters respectively decrease and increase at the end of discharge, returning to values of similar size to those observed in the as made electrode coatings. The same trend is observed for both the initial formation cycle and the cycle proceeding the fast rate cycling, however the magnitude of the changes in lattice parameters vary for the different cathodes. The largest expansion in the *c*-axis is observed for SB25, which displays a ∼0.128 Å increase at the end of the first charge cycle, compared to 0.095 Å and 0.074 Å in NMC and B25. These results are also consistent with the change in the (003) peak positions (Table S5), with SB25 and NMC demonstrating a larger low angle shift than B25.

The *c-*axis variation can also be understood by the d*Q*/d*V* (Fig. 7g and h) which show the redox peak attributed to the H2–H3 transition is shifted to higher voltage in SB25 relative to the undoped NMC, whilst a broader incomplete peak is observed for B25. The absence of this defined peak along with displaying the smallest overall change in the *c*-parameter in the first cycle is consistent with a suppression of the H2–H3 phase transition in the single boron doped material.

Additionally, no distinct splitting of the (003) peak is observed in the *ex situ* XRD of the cathodes at 4.3 V during the first formation cycle or after the fast rate cycling. Such peak splitting has previously been reported at this voltage for LNO and Ni-rich NMC due to the co-existence of the H2 and H3 phases.^3,64,65^ Instead, all the cathodes here display peak broadening in the collected XRD at the end of charge, which suggests a partial transition.^66^ Following the phase transition the (003)_H3_ peak is expected to be shifted back to a higher angle beyond that of its starting position due to the accompanying *c*-axis collapse. However, the (003) peaks in the cathodes at 4.3 V are still shifted to lower angles than in the as made electrodes, whilst the *c*-parameter is also larger, suggesting the average structure at this voltage most closely represents the H2 phase. Although the *ex situ* XRD results do not detect the onset of the H2–H3 phase transition, likely due to the relaxation of the partially formed H3 lattice, the relative peak positions and *c-*parameter in each cathode can give an indication of the extent of the phase transition.

After the fast rate cycling, the expansion in the *c*-axis and contraction in the *a*-axis at the end of charge are smaller suggesting all cathodes show some irreversible structure change from the 20 cycles at 500 mA g^−1^. Notably, the *c*-axis at the end of charge is almost unchanged for the undoped NMC material in the aged cell compared to the first cycle, whilst the *c*-axis in the doped materials display a smaller extension after cycling. However, the large reversible change in the *a*-parameter is still observed for the SB25 material after cycling.

The volume of the unit cell decreases during charge and increases during discharge as is consistent with the literature.^43,67,68^ Between the end of charge and end of discharge in the first cycle, the doped materials show a larger volume change compared to the undoped NMC, with a 2.79% and 2.23% volume increase observed in SB25 and B25, whilst only a 1.25% decrease for NMC. After cycling, this volume change decreases slightly in NMC and SB25, but considerably in B25, which shows the lowest volume change of 0.74%.

**Fig. 8:**
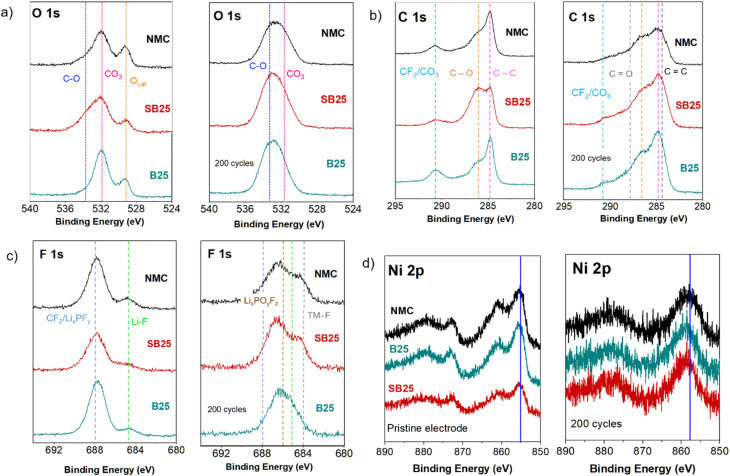
XPS collected on the pristine and cycled electrodes after 200 cycles at 200 mA g^−1^ for the C 1s, O 1s, F 1s, and Ni 2p spectra (a)–(d).

To investigate the effect of active material doping upon stabilisation of the electrode surface composition during cycling, post-mortem XPS was used to characterise the as made NMC electrodes and the same electrodes after 200 cycles at a rate of 200 mA g^−1^. Changes in the O 1s, C 1s, and F 1s spectra can be studied to investigate electrolyte decomposition and CEI evolution. Fig. 8 shows the XPS spectra for these core levels of the three pristine and cycled electrodes. Initially, the O 1s spectra show three peaks in the electrodes at 533.7 eV, 532.0 eV and 529.3 eV. The first of these likely relates to C–O from the oxidation of the conductive additive or electrolyte solvent and the presence of LiPOF species. The following peaks at lower binding energies are typically assigned to the lithium carbonate residual surface species and the lattice oxygen from the active material respectively.^69,70^ In all samples, the carbonate CO_3_/C=O peak is the dominating contribution in the spectra, highlighting the significant surface sensitivity of the Ni-rich electrodes even when prepared in a dry room. Post-mortem electrode analysis of the O 1s spectra confirms the formation of a substantial surface layer on the cathodes, reducing the relative proportion of the lattice oxygen to below 1% in each of the samples. Within the surface species, the proportion of C–O is largest for the baseline NMC, which has previously been ascribed to an increase in polyether products from severe electrolyte decomposition.^69,71^ The proportion of this peak in the O 1s spectra is reduced in the SB25 sample and much smalller still in the B25, which could suggest a lower level of electrolyte decomposition for the boron doped material, although a much larger number of different scan spots would be needed to increase confidence in this.

Prior to cycling, the C 1s spectra of the electrodes display three main features centred around peaks at 284.9 eV (C–C), 286.3 eV (C–O), and 290.4 eV (CO_3_/CF_2_), whilst a C–H environment is also typically present around 285.5 eV but is convoluted with other peaks which results in peak shape changes, and are not labelled specifically. Additionally, the cycled electrodes all show an increase in the peak intensity ratio C–O/C–C, which is associated with the decomposition of the electrolyte and subsequent build-up of CEI species.^72^ Compared with the undoped NMC, B25 and SB25 both display smaller proportion of C–O relative to the C–C, indicating the electrolyte decomposition is somewhat reduced for the doped materials. This result is also consistent with the trend in the electrochemical cycling performance of each material, in which B25 demonstrates the highest capacity retention.

Fig. S6 shows the Li 1s spectra of the pristine and aged electrodes. Prior to cycling, a single peak in the Li 1s spectra is present in each of the cathode electrodes at 55.2 eV which is present again at the same position in the cycled electrodes. However, an additional peak can be detected in the cycled NMC baseline electrode, situated at a higher binding energy of 57.7 eV. The occurrence of this second peak in the baseline NMC hints at the formation of a larger proportion of LiF, which has been linked to the decomposition of LiPF_6_-based electrolytes, or the irreversible consumption of active Li in the CEI.^73^ The absence of this peak in the Li 1s spectra of SB25 and B25 suggests less degradation, which correlates with the lower capacity fade observed during cycling.

The F 1s spectra of the electrodes prior to cycling all show two peaks, the larger one situated at around 688 eV mainly arising from the C–F in the PVDF binder, whilst the smaller peak at 685 eV relates to LiF.^73^ Following cycling, the peak shape becomes more convoluted with the appearance of additional peaks located at 686.4 eV and 684.3 eV in contrast to the greatly reduced intensity of the PVDF feature. This new peak shape is consistent with the additional presence of Li_*x*_PO_*y*_F_*z*_ from the electrolyte decomposition (∼686 eV) and transition metal fluorides from the reaction of surface migrated TM and the electrolyte (∼684 eV) respectively.^74^ The TM-F species is notably less dominant in the peak shape of the cycled B25 electrode, suggesting a smaller proportion of the insulating transition metal fluoride is present in its CEI layer. For the Ni 2p spectra, the main 2p_3/2_ and 2p_1/2_ peaks appear at ∼855.5 eV and ∼873.0 eV in the as made electrodes of each cathode, but following cycling the centre of the Ni 2p_3/2_ peak is situated slightly higher, at around 857.7 eV for the pristine NMC and at 858.5 eV in both SB25 and B25. This peak shift to higher binding energy has previously been attributed to a transformation of the Ni surface, for example, to NiF_2_ or surface reconstruction to a rock-salt structure.^75,76^

### 3.5 *Ex situ* XAS

**Fig. 9:**
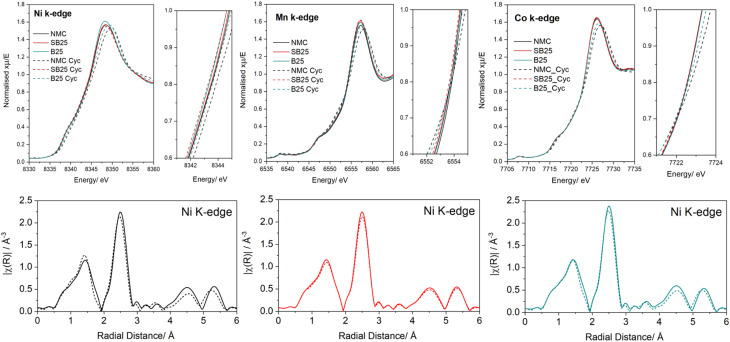
Normalised XANES spectra of the pristine and cycled electrode of NMC, SB25, and B25 at the Ni, Co, and Mn K-edges. EXAFS spectra of the cathode materials at the Ni K-edge.

The variation in transition metal oxidation state after cycling was further studied by measuring XANES on spectra of the Ni, Mn, and Co K-edges on pristine electrodes and electrodes extracted from cells following 200 charge and discharge cycles, which are depicted in Fig. 9. As seen previously in the powder samples (Fig. 3), the white line position of all the electrode samples in the Ni K-edge are shifted to higher energies compared to the Ni^2+^ standards by the same gap present in the powders, suggesting no additional oxidation of the vacuum stored electrodes in the dry room. After 200 cycles, the white line energy of the pristine NMC at the Ni edge is shifted by 1.4 eV to a higher energy position, whereas a smaller shift of 1.1 eV is observed for B25, whilst no shift occurs for SB25. The same trend is also displayed in both the Co and Mn K-edges, where the positive energy shifts are 0.9 eV and 0.8 eV respectively for the baseline NMC, which are reduced to 0.3 eV and 0.5 eV for B25, whilst the peak intensity maximum positions for SB25 remain constant in the pre-cycled and cycled electrode. The larger energy shifts in the cycled baseline NMC indicates an increase in average oxidation states of the transition metals, whereas this is stabilised by the inclusion of boron in the structure acts, whilst the addition of tin appears to indicate complete retention of the TM valences. Therefore, after the 200th cycle the doped materials demonstrate a higher reversibility of the electrochemical active redox reactions, which is supported by the higher capacity retentions displayed in these cathodes.

Alongside the energy shift, changes in the loss of white line intensity are also observed in the Ni K-edge spectra for all cathodes, although the exact loss varies between the samples. Compared with the pristine NMC, the peak intensity loss is reduced for SB25, suggesting the co-doping stabilises the local structure environment of the Ni during cycling. The opposite effect is actually observed for B25, however the peak intensity in the cycled boron doped electrode is still higher than that displayed for the corresponding aged NMC electrode. The effect of doping on the peak intensity change after cycling in the Co and Mn K-edges is more aligned, with SB25 and B25 both acting to reduce the decay in peak intensity. Notably, the Mn K-edge spectra of SB25 before and after cycling can be overlaid almost identically, suggesting the structure and valence of Mn in SB25 is virtually unchanged after 200 charge and discharge cycles. Furthermore, the baseline NMC cathode shows a larger increase in intensity of the Mn pre-edge peak compared to the doped materials, which indicates a larger disorder in the octahedral coordination.^77^

The Ni EXAFS spectra of the cathodes presented in Fig. 9 display little variation in the electrodes after cycling for the main scattering peaks of Ni–TM and Ni–O located below 3 Å. Each cathode demonstrates a similar small loss of intensity at the Ni–TM peak, whilst no change in the apparent radial distance is observed following cycling. The change in the peak intensity of the Ni–O shell reveals more variation between samples, with the intensity increasing for the NMC baseline, but decreasing in SB25 and remaining unchanged in B25. A small shift in the Fourier transform position is also observed for the Ni–O shell in the undoped NMC material after cycling. This shift could indicate a reduction in bond length, which would be consistent with the higher average oxidation state of Ni observed in the XANES, further fitting and phase corrections are required for precise quantification.

The improved performance of SB25 relative to B25 and undoped NMC arises from the convergence of three different impacts of the dopant addition:

1. Suppression of the H2–H3 phase transition and microcrack formation.

The d*Q*/d*V* plots in Fig. 6 show that the high-voltage H2–H3 peak, which reflects the destructive anisotropic contraction of the *c*-axis at high levels of delithiation, declines in intensity far less rapidly in SB25 over 100 cycles than in the undoped NMC. This is consistent with the pillaring action of the isovalent Sn^4+^ dopant which stabilises the transition metal layer to a greater degree against the large volume changes associated with the H2–H3 transition. The *ex situ* XRD data in Fig. 7 further quantify this; SB25 exhibits a *c*-axis expansion of ∼0.128 Å at end of charge during the first cycle, compared to 0.095 Å for NMC and 0.074 Å for B25, confirming that the Sn dopant expands and stabilises the Li slab spacing. The resulting capacity retentions after 100 cycles at 200 mA g^−1^ are 88.7% (SB25), 92.7% (B25), and 78% (NMC), demonstrating that both dopants substantially extend cycle life relative to the baseline.

2. Reduction of charge-transfer resistance and improvement of Li-ion transport kinetics, especially at low temperature.

The EIS results (Fig. 5 and Tables S2–S4) show that at room temperature, RCT at 50% SOC is reduced from 10.34 Ω (NMC) to 8.79 Ω (SB25), with B25 intermediate at 10.04 Ω. This improvement is significant at −5 °C, where the pristine NMC develops a second, dominant charge-transfer process (RCT reaching 717 Ω at 4.3 V), while SB25 exhibits a single, substantially lower charge-transfer arc (*R*_CT_ = 86.5 Ω at 50% SOC, declining to 71.4 Ω at 4.3 V). The elimination of the second semi-circle in the doped materials, which is suppressed in B25 and absent entirely in SB25, indicates that the dopants prevent the buildup of a highly resistive interfacial layer at low temperature. The corresponding exchange current densities (Fig. 5f) confirm that SB25 maintains the highest *J*_0_ across the full delithiation window at −5 °C, while B25 shows a plateau at a lower value and NMC follows an inverted V-shape with a narrow maximum of 0.166 A m^−2^. The GITT diffusion data (Fig. S4) show that SB25 consistently displays larger *D*_Li^+^_ values across the full voltage window at −5 °C, most notably between 3.8 V and 4.3 V, consistent with the wider Li slab spacing promoting faster solid-state diffusion at the temperature where transport kinetics are most limiting.

3. Suppression of CEI degradation and transition-metal fluorination at the electrode surface.

The post-mortem XPS data (Fig. 8 and S6) show that the undoped NMC develops a second Li 1s peak at 57.7 eV after 200 cycles, consistent with significant LiF accumulation from LiPF_6_ decomposition, which is absent in both B25 and SB25. The F 1s spectra confirm that the transition-metal fluoride species (∼684 eV) is notably less pronounced in the cycled B25 electrode. The C 1s spectra show that the relative proportion of C–O (associated with polyether electrolyte decomposition products) is lower in both doped materials. Together, these findings indicate that the chemically stable dopant-modified surface, whether the Li–B–O surface species observed in the B 1s XPS at 190.8–191.5 eV, or the Sn^4+^-reinforced transition metal layer, reduces parasitic electrolyte oxidation and CEI thickening, which is the dominant degradation mechanism at elevated temperature (45 °C).

The synergy between Sn and B, specifically at low temperature, arises because these three effects converge in SB25 in a way that neither single dopant achieves alone. B25 suppresses H2–H3 transitions and CEI growth effectively (reflected in its high 92.7% capacity retention) but shows poor rate capability at −5 °C (only 42.4% of initial capacity retained at 500 mA g^−1^), likely because the B-associated lattice effects do not sufficiently widen the Li slab for fast low-temperature diffusion. SB25 delivers both the structural stabilisation and the kinetic benefit simultaneously: a 25% improvement in specific capacity at 500 mA g^−1^ relative to NMC at −5 °C, a 56.2% rate capacity retention *vs.* 42.4% for B25 at the same conditions, and the highest 99% capacity recovery after returning to slow rate cycling.

## 4 Conclusions

This work demonstrates that boron (B25) and tin-boron (SB25) co-doping of Ni-rich NMC9055 cathodes significantly enhance electrochemical performance and structural stability, particularly across a technologically relevant temperature range of −5 °C to 45 °C. Both dopants integrate into the α-NaFeO_2_ structure as confirmed by Rietveld refinements. They exhibit expanded lattice parameters and reduced cation mixing, particularly in SB25. The location of Boron is not conclusive, but it is likely both incorporated into the bulk *via* an interstitial sites, as well as forming a boron rich layer at the surface. Sn^4+^ substitution on the transition metal site acts as a structural pillar, suppressing of the detrimental H2–H3 phase transitions that drives the anisotropic *c*-axis contraction and microcrack formation at high states of charge.

The electrochemical benefits of co-doping are most pronounced at low temperatures, where Li-ion transport kinetics are the dominant bottleneck. At −5 °C, pristine NMC develops a second, dominant charge-transfer process with *R*_CT_ reaching 717 Ω at 4.3 V, whereas SB25 exhibits a single charge-transfer arc with *R*_CT_ = 86.5 Ω at 50% SOC, declining to 71.4 Ω at full charge. The corresponding rate performance at 500 mA g^−1^ at −5 °C is 91 mAh g^−1^ for SB25 *versus* 73 mAh g^−1^ for pristine NMC, which is a 25% improvement, while B25 delivers only 42% of its initial rate capacity under the same conditions. This confirms the specific role of Sn in widening the Li-ion diffusion pathway. GITT measurements confirm that SB25 maintains larger Li-ion diffusion coefficients across the full voltage window at −5 °C, most notably during charging between 3.8 V and 4.3 V. At room temperature, co-doping reduces *R*_CT_ at 50% SOC from 10.34 Ω (NMC) to 8.79 Ω (SB25) and increases the average diffusion coefficient from 1.50 to 1.62 × 10^−12^ cm^2^ s^−1^. At 45 °C, both doped materials sustain higher exchange current densities than pristine NMC across the full charge window, with average *J*_0_ values of 3.6 A m^−2^ (SB25) and 3.5 A m^−2^ (B25) *versus* 2.6 A m^−2^ (NMC).

Long-term cycling at approximately 1C reveals that both dopants substantially mitigate capacity fade, with retentions of 92.7% (B25) and 88.7% (SB25) after 100 cycles compared to 78% for pristine NMC. The d*Q*/d*V* data show that the H2–H3 peak, which declines sharply in the undoped material over 100 cycles, is far better preserved in SB25, confirming structural stabilisation throughout extended operation. Post-mortem XPS after 200 cycles show that pristine NMC develops a LiF-rich second Li 1 s component at 57.7 eV and a prominent transition-metal fluoride peak at 684 eV, both of which are substantially suppressed in B25 and absent in SB25. Post-mortem XAS confirms that after 200 cycles the Ni K-edge shifts by 1.4 eV in pristine NMC, by 1.1 eV in B25, and by 0 eV in SB25, with Mn coordination virtually unchanged in the co-doped material, together indicating that Sn–B co-doping stabilises both the bulk oxidation state and the local coordination environment of the transition metals throughout extended cycling.

In conclusion, Sn–B co-doping synergistically enhances the high-rate and temperature-dependent performance of Ni-rich NMC955 cathodes. The improved performance, particularly at low temperatures, underscores the potential of tailored doping strategies for advancing lithium-ion batteries for electric vehicles and other demanding applications.

## Conflicts of interest

There are no conflicts to declare.

## Supplementary Material

TA-014-D6TA01388K-s001

## Data Availability

The data supporting the findings of this study are available from the corresponding authors upon reasonable request. Supplementary information (SI): all relevant experimental data, including electrochemical measurements, XRD refinements, XPS, XAS, and impedance analysis datasets, are provided within the article and its supporting information. Raw datasets and additional processed data generated during the current study are available from the authors on request. See DOI: https://doi.org/10.1039/d6ta01388k.
