# Supplementary material for: Improved cycle life and Li-ion transport parameters at low temperature in doped Ni-rich NMC cathodes
Source: J Mater Chem A Mater. 2026 Apr 23;14(34):22411–28. doi: 10.1039/d6ta01388k (PMC13123467; doi:10.1039/d6ta01388k)
Supplement: TA-014-D6TA01388K-s001 [file TA-014-D6TA01388K-s001.pdf]

Supplementary Information (SI) for Journal of Materials Chemistry A.
This journal is © The Royal Society of Chemistry 2026

Supporting Information

Improved Cycle Life and Li-Ion Transport Parameters at Low Temperature in Doped Ni-Rich NMC Cathodes

Ethan Williams,[a,c] David Burnett,[a,c] Wilgner Lima da Silva,[b,c] Jack E. N. Swallow,[d] Ryan Parmenter,[e] Robert S. Weatherup,[c,d] Peter Slater,[b,c] and Emma Kendrick.[a,c,*]*

[a] *School of Metallurgy and Materials, University of Birmingham, Edgbaston, Birmingham, B15 2TT, UK*

[b] *School of Chemistry, University of Birmingham, Edgbaston, Birmingham, B15 2TT, UK*

[c] *The Faraday Institution, Quad One, Becquerel Avenue, Harwell Campus, Didcot, OX11 0RA, UK*

[d] *Department of Materials, University of Oxford, Parks Road, Oxford OX1 3PH, United Kingdom*

[e] *School of Physical Sciences, University of Kent, Canterbury, Kent CT2 7NH, UK*

**Corresponding author(s): e.kendrick@bham.ac.uk and etw078@student.bham.ac.uk*

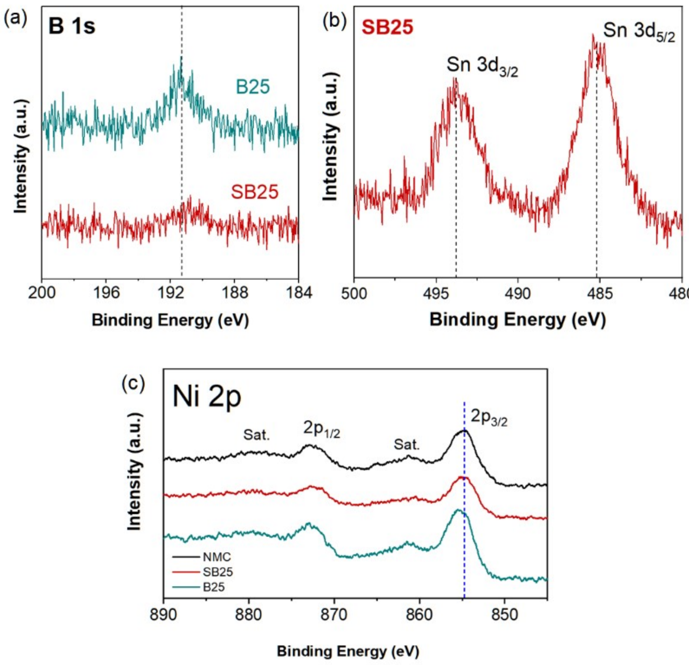

Figure S1: XPS spectra of the baseline and doped cathode powder materials, showing the (a) B 1s, (b) Ni 2p, and (c) Sn 3d regions.

Table S1: The threshold energies (E_0) of the main edge of XANES Ni, Mn, and Co K-edge spectra for the powdered cathode and reference samples. [Ac] = $CH_3CO_2^{2-}$

**Ni K-edge**	**E_0/ eV**	**Mn K-edge**	**E_0/ eV**	**Co K-edge**	**E_0/ eV**
NMC	8347.95	NMC	6556.42	NMC	7726.66
B25	8347.80	B25	6556.37	B25	7726.62
SB25	8347.88	SB25	6556.46	SB25	7726.55
NiO	8345.53	$Mn[Ac]_2$	6547.97	$Co[Ac]_2$	7721.53
$Ni[Ac]_2$	8345.92	Mn_2O_3	6552.56	Co_3O_4	7724.62
		MnO_2	6555.28	$LiCoO_2$	7725.70

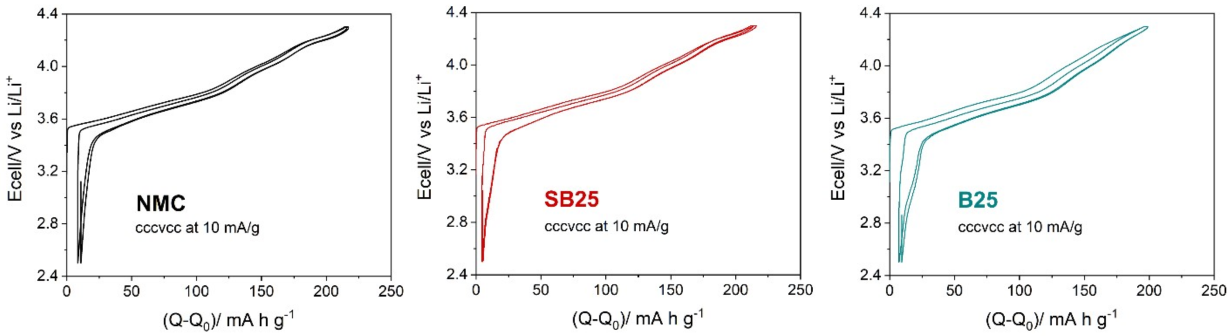

Figure S2: Formation profiles of the 1st and 2nd charge-discharge cycles of NMC, SB25, and B25 measured at 10 mA g^{-1} between 4.3 – 2.5 V.

Table S2: Equivalent circuit element values taken from the fitted EIS results at different SOC recorded at room temperature.

	NMC				SB25			B25		
SOC %	R_S	R_{SEI}	R_{CT}	R_{CT}	R_S	R_{SEI}	R_{CT}	R_S	R_{SEI}	R_{CT}
0	3.08	7.70	11.0	11.6	1.32	30.42	208	1.16	67.49	274.4
10	4.35	7.71	6.70	27.0	1.62	2.23	19.4	1.37	3.21	19.3
20	3.45	4.30	8.83	9.58	1.55	1.63	14.7	1.38	2.46	15.0
30	3.60	5.77	4.95	7.74	1.60	1.49	13.2	1.50	2.41	12.1
40	4.33	2.23	5.36	9.04	1.05	4.57	8.84	1.48	2.54	9.93
50	3.76	4.48	4.32	7.48	1.13	3.31	8.79	1.42	2.04	10.0
60	4.27	3.19	4.54	8.07	1.67	1.13	9.59	1.48	1.85	9.05
70	3.94	3.18	4.59	9.82	1.10	2.48	8.71	1.41	1.79	8.47
80	3.28	3.90	4.22	17.4	1.17	2.07	8.40	1.45	1.69	7.70
90	3.79	4.16	4.41	53.1	1.58	0.88	8.16	1.46	1.70	7.25
100	3.08	7.70	11.0	11.6	1.36	1.09	10.44	1.40	1.83	6.91

Table S3: Equivalent circuit element values taken from the fitted EIS results at different SOC recorded at 45°C

	NMC			SB25			B25		
SOC %	R_S	R_{SEI}	R_{CT}	R_S	R_{SEI}	R_{CT}	R_S	R_{SEI}	R_{CT}

0	1.42	7.66	17.5	1.34	2.31	8.79	1.433	1.58	12.1
10	1.56	4.47	9.45	1.41	1.74	7.30	1.515	2.77	8.10
20	1.19	3.24	8.76	1.30	1.81	6.54	1.322	0.632	10.4
30	1.18	6.32	7.72	1.30	1.80	5.83	1.310	0.540	9.91
40	1.04	5.60	6.51	1.35	1.34	5.97	1.494	3.86	5.33
50	0.791	5.88	5.94	1.39	1.32	5.50	1.463	3.82	4.51
60	0.833	5.39	5.64	1.34	1.34	5.27	1.556	2.55	5.23
70	0.834	5.38	5.98	1.33	1.44	4.90	1.372	3.54	3.72
80	0.867	5.30	6.06	1.31	1.50	4.81	1.660	1.03	5.81
90	0.963	5.97	6.50	1.32	1.25	5.14	1.522	2.02	4.61
100	1.05	6.23	20.2	1.29	1.23	5.29	1.538	1.27	5.49

Table S4: Equivalent circuit element values taken from the fitted EIS results at different SOC recorded at -5℃

	NMC				SB25			B25		
SOC %	R_S	R_{SEI}	R_{CT}	R_{CT}	R_S	R_{SEI}	R_{CT}	R_S	R_{SEI}	R_{CT}
0	2.58	12.1	98.8	-	2.72	7.82	1530	3.37	15.4	419
10	2.67	18.3	75.9	346	1.94	11.04	154	3.22	13.1	236
20	2.73	17.3	64.6	132	1.88	8.24	116	3.34	11.6	177
30	2.70	16.8	58.7	100	1.32	8.61	101	3.29	11.8	183
40	2.65	16.5	55.8	115	1.03	8.46	91.5	3.26	11.7	182
50	2.56	16.2	50.7	136	1.19	7.46	86.5	3.23	11.4	172
60	2.51	15.7	51.2	161	1.55	6.20	84.3	3.23	11.7	190
70	2.40	15.5	50.1	305	2.09	4.87	80.0	3.20	11.3	173
80	2.36	15.7	51.2	348	1.06	6.91	74.7	3.28	11.9	216
90	2.36	15.5	50.5	356	1.02	6.98	76.7	3.26	11.8	210
100	2.33	15.4	49.8	717	0.86	7.22	71.4	4.62	7.01	232

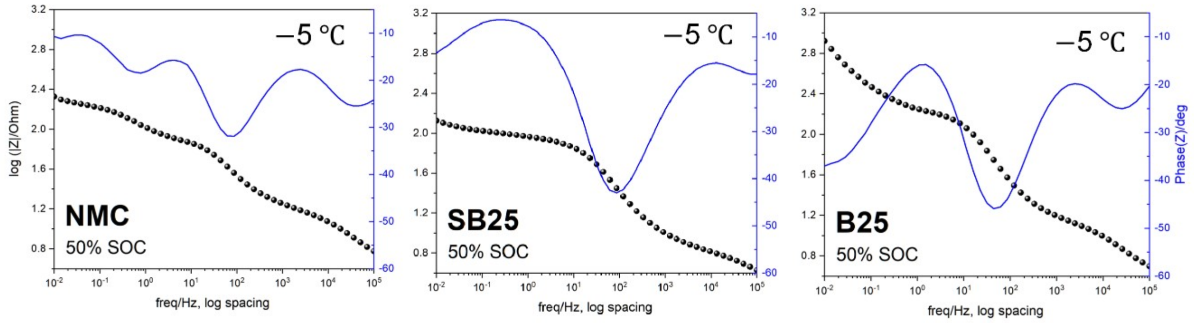

Figure S3: Bode plots from EIS measurements in cathode half-cells recorded at 50% SOC tested at -5℃.

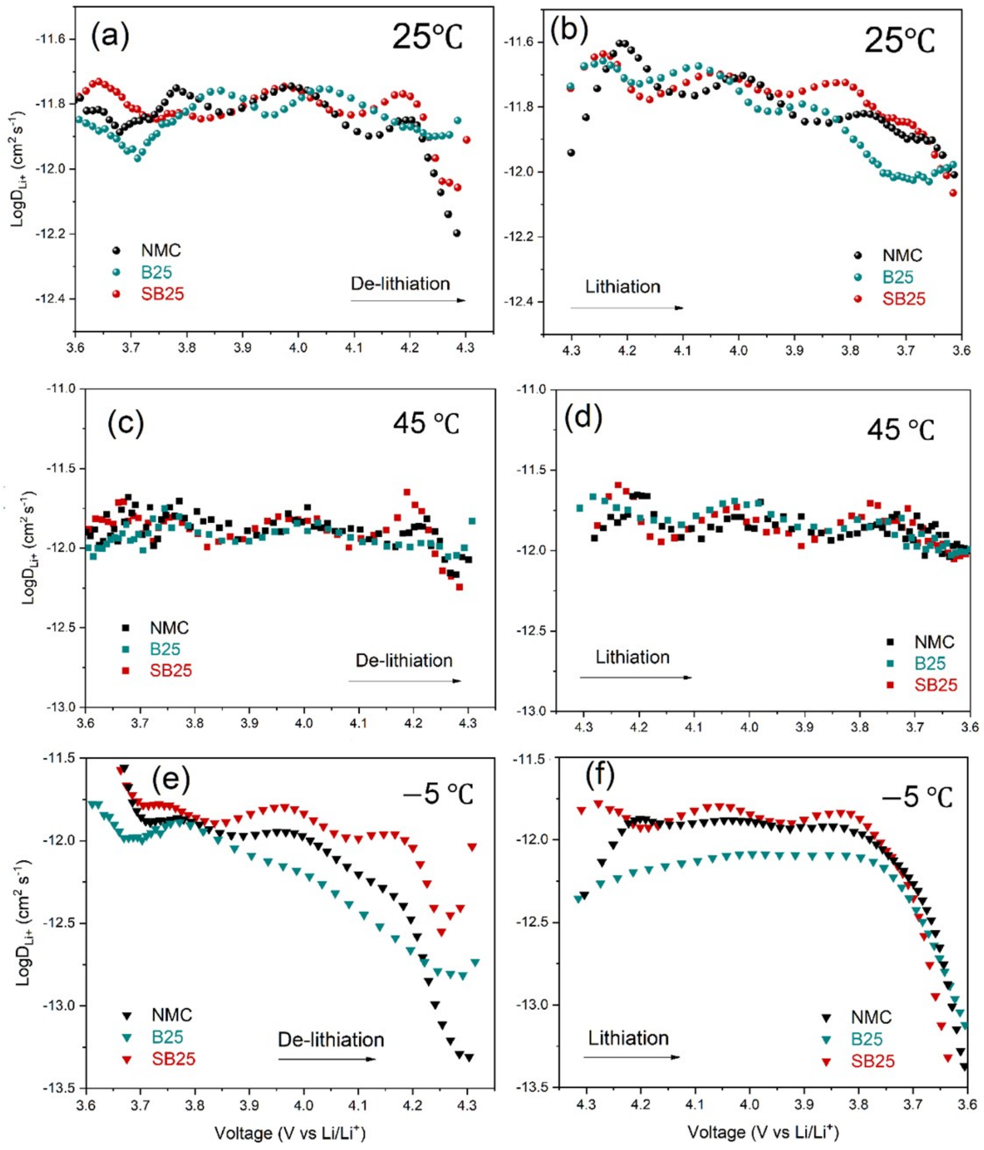

Figure S4: GITT results showing the log of the diffusion coefficients during charge (a, c, d) and discharge (b, d, f) at room temperature (a, b), 45 ℃ (c, d), and − 5℃ (e, f).

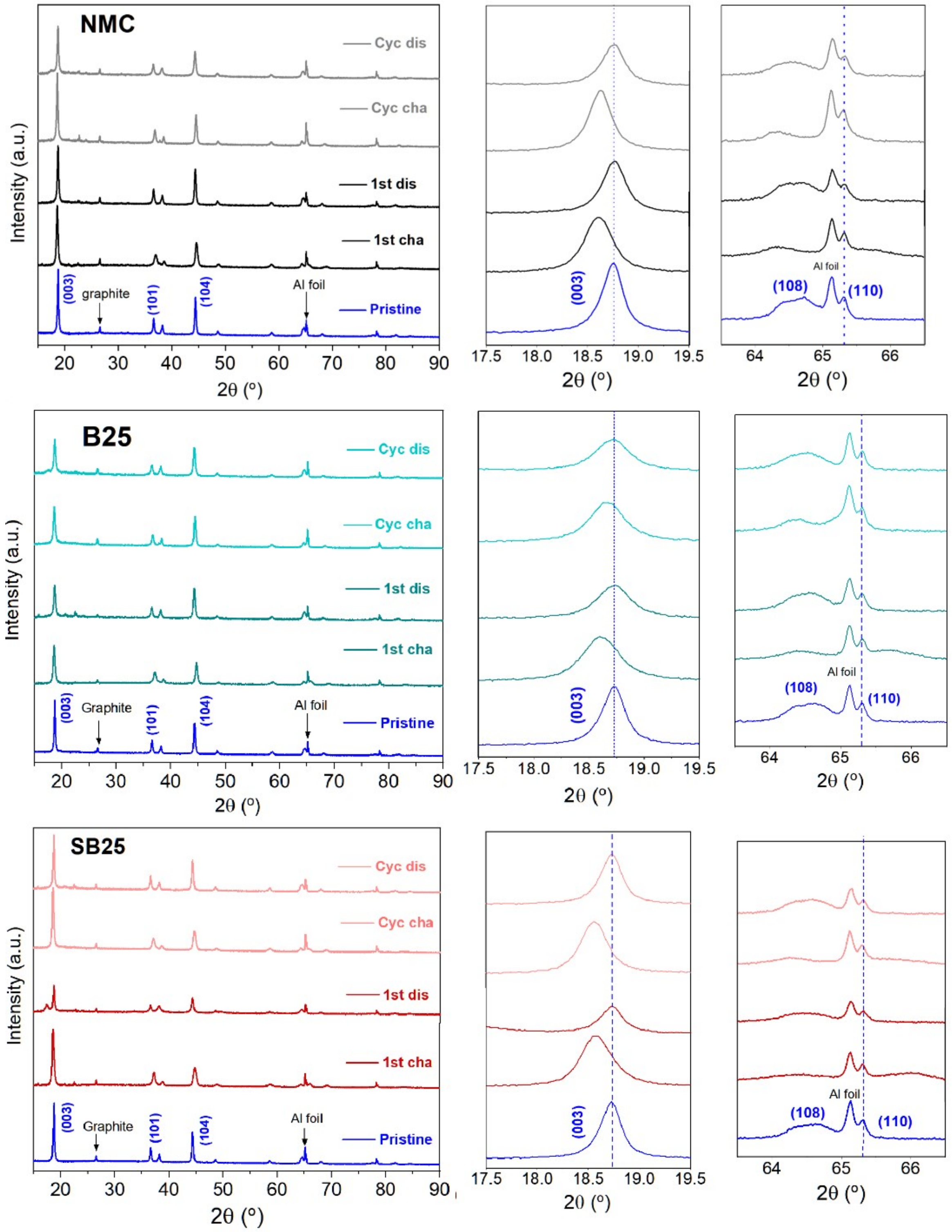

Figure S5: Ex-situ XRD patterns collected on electrodes from cells cycled between 4.3 – 2.5 V at 10 mA g^{-1} during the first formation cycle and in after 20 cycles at a discharge rate of 500 mA g^{-1}.

Table S5: Peak positions of the (003) reflection in the pristine electrode and electrodes in the charged state during the 1st cycle and after 20 cycles at 500 mA g^{-1}.

Cathode	(003) 2θ position/ °			$\Delta(003)_{\text{Pristine – 4.3 V}}$	
	Pristine	1st (4.3 V)	Cyc. (4.3 V)	1st	Cyc.
NMC	18.763	18.612	18.631	-0.151	-0.132
B25	18.726	18.593	18.669	-0.133	-0.057
SB25	18.726	18.574	18.555	-0.152	-0.171

Table S6: Lattice parameters calculated from Rietveld refinements of the as made NMC, B25, and SB25 pristine electrodes and ex-situ XRD measurements of these electrodes at the end of charge (4.3 V) and end of discharge (2.5 V) of the 1st formation cycle and after 20 cycles at 500 mA g^{-1}.

**Cathode**	**Sample**	**a/ Å**	**c/ Å**	**Volume/ Å^3**	**Rwp %**
NMC	Pristine	2.877486	14.17593	101.65	12.76
NMC	1st Cha	2.850072	14.27104	100.392	14.00
NMC	1st Dis	2.875671	14.1933	101.646	8.41
NMC	Cyc Cha	2.85847	14.26823	100.964	15.80
NMC	Cyc Dis	2.880743	14.20206	102.068	14.41
B25	Pristine	2.880283	14.19949	102.017	10.90
B25	1st Cha	2.842672	14.27468	99.897	10.15
B25	1st Dis	2.881105	14.20655	102.126	11.04
B25	Cyc Cha	2.866755	14.25965	101.489	10.89
B25	Cyc Dis	2.884258	14.19168	102.243	12.35
SB25	Pristine	2.880686	14.18203	101.92	16.17
SB25	1st Cha	2.834774	14.30991	99.587	10.30
SB25	1st Dis	2.882279	14.2288	102.37	15.09
SB25	Cyc Cha	2.840289	14.27736	99.748	13.79
SB25	Cyc Dis	2.881046	14.21989	102.218	9.80

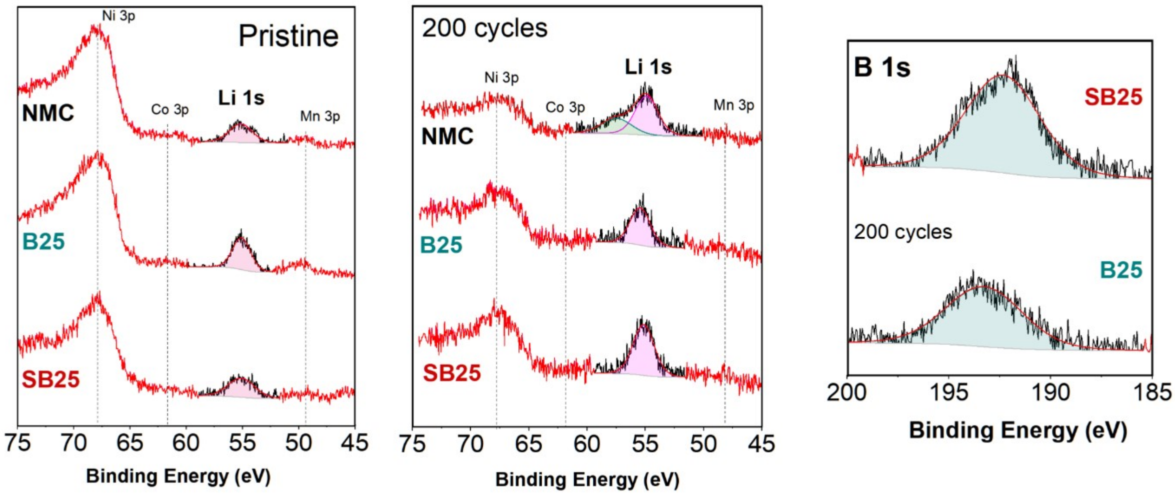

Figure S6: XPS results showing the Li 1s spectra of the pristine and aged NMC cathode electrodes, and the B 1s spectra of the SB2 and B25 cycled electrodes.

Table S7 Summary of doped high nickel NMC materials, and their electrochemical performance properties

Material	Dopant / strategy Mol %	Voltage (V)	Retention	Cycles (rate)	Initial cap. mAh g^{-1}	Rate cap. (500 mA g^{-1})	T tested °C	Ref.
NMC955 this work	Sn–B co-doped 2.5% (SB25)	2.5–4.3	88.7%	100 (1C)	210	157 mAh g^{-1} (45 °C) 91 mAh g^{-1} (−5 °C)	−5, 25, 45	—
NMC955 this work	B-doped 2.5% (B25)	2.5–4.3	92.7%	100 (1C)	191 [1]	140 mAh g^{-1} (45 °C) 42% retention (−5 °C)	−5, 25, 45	—
NMC955 this work	Undoped NMC	2.5–4.3	78%	100 (1C)	208	133 mAh g^{-1} (45 °C) 73 mAh g^{-1} (−5 °C)	−5, 25, 45	—
NMC955 (NCM90)	B-doped 1	3.0–4.3	91%	100 (0.5C, 55 °C)	237	Not reported	55 °C only	1
$LiNi_{0.92}Co_{0.04}Mn_{0.04}O_2$	B Doped 1 P Doped 1	3.0–4.4	80% 75%	100 0.3C	225	170 mAhg^{-1} @2C	RT only	2
NCA95 ($LiNi_{0.95}Co_{0.04}Al_{0.01}O_2$)	B-doped 1.5	3.0–4.3	88%	100 (0.5C)	~230	Not reported	30 °C	3
NCM90 ($LiNi_{0.9}Co_{0.1}O_2$)	B-doped 0.5 Mo-doped 0.5 B-Mo 0.25-0.25	3.0–4.3	80% 95% 90%	100 (1C)	234	Not reported	RT only	4
NMC811	Ti-doped (bulk + surface) 0.9%	2.8–4.3	91.5%	100 (C/3)	~190	~155 @3C	RT only	5
NMC811	Zr-doped 0.1 mol% (co-precip.)	3.0–4.4	85%	100 (1C)	205	150 @5C	RT only	6

Note: direct quantitative comparisons are limited by differences in voltage window, current density, cell format and temperature. Capacity retention values for this work are quoted after 100 cycles at ~1C (200 mA g^{-1} discharge). "RT" = room temperature. Rate capability at 500 mA g^{-1} at −5 °C and 45 °C is a distinguishing feature of the present work not reported in any of the listed references.

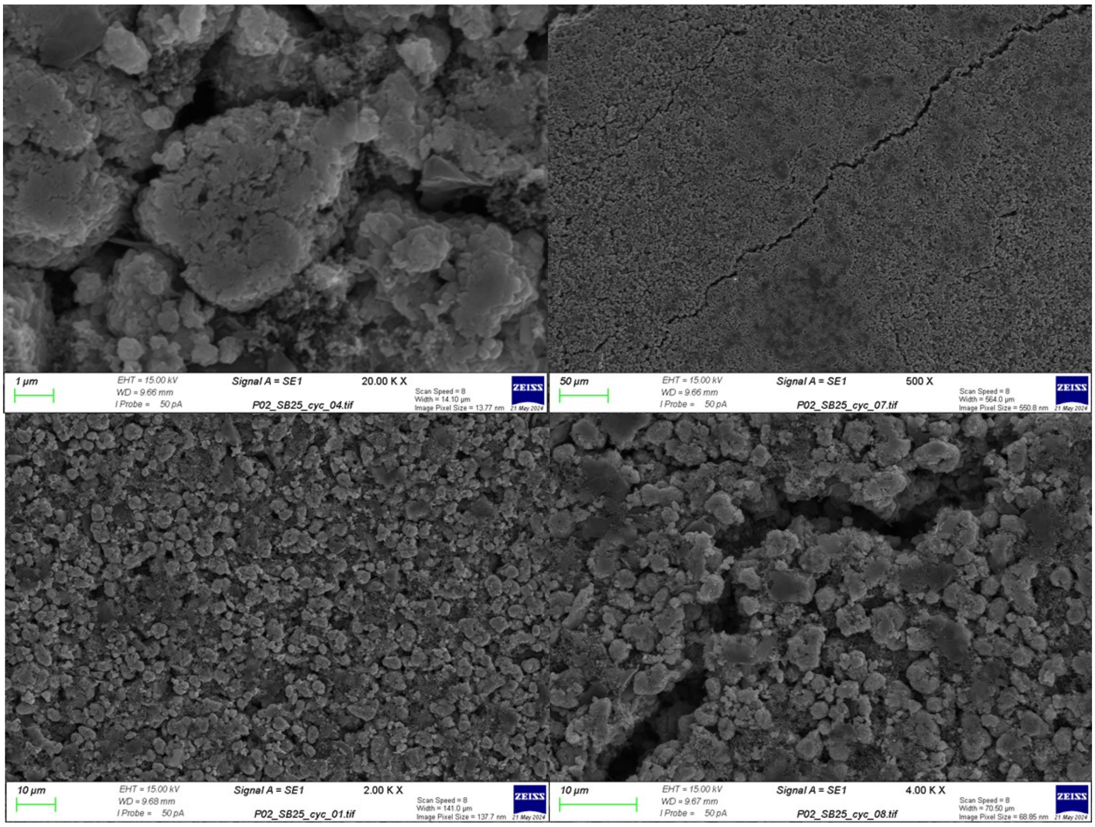

Figure S7: SEM images of the NMCSB25 from a cycled single layer pouch cell vs graphite, 200 cycles at 200 mA/g

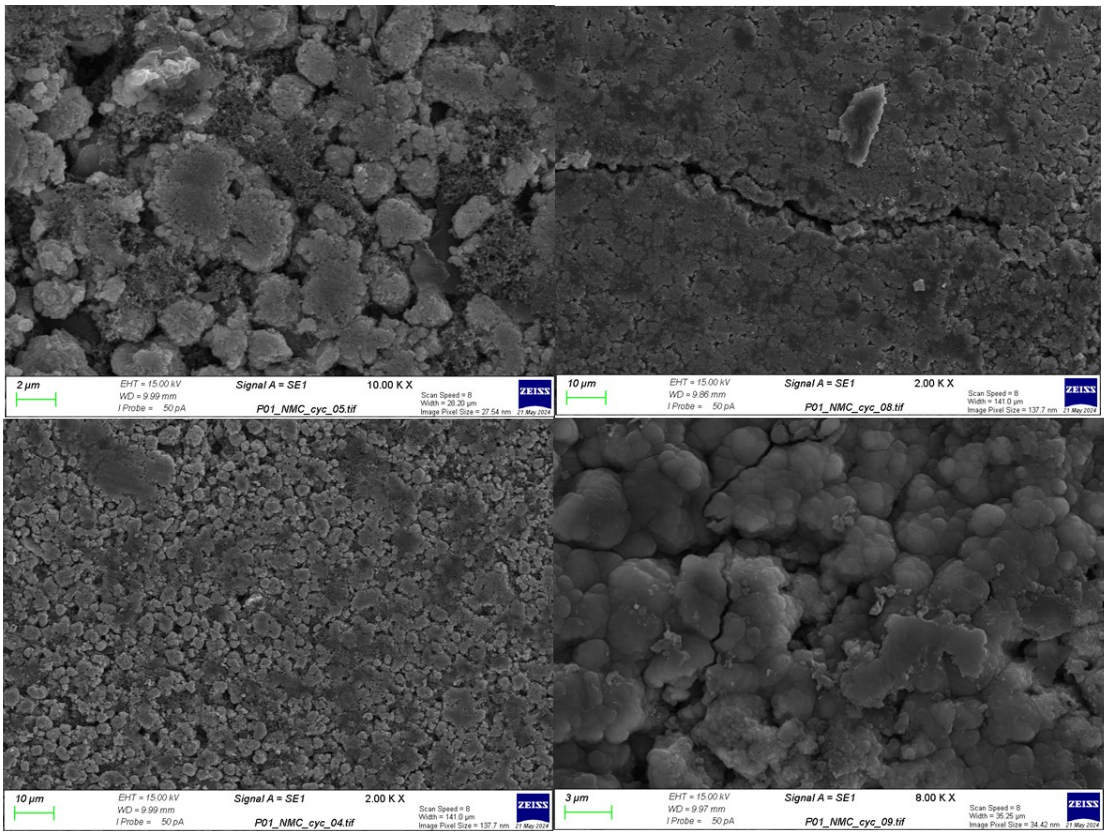

Figure S8: SEM images of the NMC, from a cycled single layer pouch cell vs graphite, 200 cycles at 200 mA/g

1 K.-J. Park, H.-G. Jung, L.-Y. Kuo, P. Kaghazchi, C. S. Yoon, Y.-K. Sun, K.-J. Park, Y.-K. Sun, H.-G. Jung, L.-Y. Kuo, P. Kaghazchi and C. S. Yoon, *Adv. Energy Mater.*, 2018, **8**, 1801202.

2 C.-H. Jung, D.-H. Kim, D. Eum, K.-H. Kim, J. Choi, J. Lee, H.-H. Kim, K. Kang, S.-H. Hong, C. Jung, D. Kim, D. Eum, K. Kim, J. Choi, J. Lee, H. Kim, K. Kang and S. Hong, *Adv. Funct. Mater.*, 2021, **31**, 2010095.

3 Y. S. Kim, J. H. Kim, Y. K. Sun and C. S. Yoon, *ACS Appl. Mater. Interfaces*, 2022, **14**, 17500–17508.

4 F. A. Susai, A. Bano, S. Maiti, J. Grinblat, A. Chakraborty, H. Sclar, T. Kravchuk, A. Kondrakov, M. Tkachev, M. Talianker, D. T. Major, B. Markovsky and D. Aurbach, *J. Mater. Chem. A Mater.*, 2023, **11**, 12958–12972.

5 F. Bizzotto, W. Dachraoui, R. Grissa, W. Zhao, F. Pagani, E. Querel, R. S. Kühnel and C. Battaglia, *Electrochim. Acta*, 2023, **462**, 142758.

6 M. Colalongo, B. Ali, I. Martens, M. Mirolo, E. Laakso, C. Atzori, G. Confalonieri, P. Kus, A. Kobets, X. Kong, T. Schulli, J. Drnec, T. Kankaanpää and T. Kallio, *ACS Appl. Mater. Interfaces*, 2024, **16**, 28683–28693.
